# Self-Powered Sensors and Systems Based on Nanogenerators

**DOI:** 10.3390/s20102925

**Published:** 2020-05-21

**Authors:** Zhiyi Wu, Tinghai Cheng, Zhong Lin Wang

**Affiliations:** 1 Beijing Institute of Nanoenergy and Nanosystems, Chinese Academy of Sciences, Beijing 100085, China; wuzhiyi@binn.cas.cn (Z.W.); chengtinghai@binn.cas.cn (T.C.); 2 School of Materials Science and Engineering, Georgia Institute of Technology, Atlanta, GA 30332, USA

**Keywords:** sensors, self-powered, triboelectric nanogenerator, piezoelectric nanogenerator

## Abstract

Sensor networks are essential for the development of the Internet of Things and the smart city. A general sensor, especially a mobile sensor, has to be driven by a power unit. When considering the high mobility, wide distribution and wireless operation of the sensors, their sustainable operation remains a critical challenge owing to the limited lifetime of an energy storage unit. In 2006, Wang proposed the concept of self-powered sensors/system, which harvests ambient energy to continuously drive a sensor without the use of an external power source. Based on the piezoelectric nanogenerator (PENG) and triboelectric nanogenerator (TENG), extensive studies have focused on self-powered sensors. TENG and PENG, as effective mechanical-to-electricity energy conversion technologies, have been used not only as power sources but also as active sensing devices in many application fields, including physical sensors, wearable devices, biomedical and health care, human–machine interface, chemical and environmental monitoring, smart traffic, smart cities, robotics, and fiber and fabric sensors. In this review, we systematically summarize the progress made by TENG and PENG in those application fields. A perspective will be given about the future of self-powered sensors.

## 1. Introduction

### 1.1. Historical Development

To the best of my knowledge, the history of sensors in China can be traced back to the Spring and Autumn period (770–476 BCE), with the time observing device called the sundial. During the Warring States period (475–221 BCE), there appeared a direction indicating device called a “sinan” (“south-pointing ladle”), made with a magnet, which is one of the four great inventions of China. The compass, born from it, opened up a maritime navigation era and promoted the development of international trade. From then on, human beings witnessed unprecedented social development. By modern times, with the progress of science and technology and under the advancement of the industrial revolution, sensor technology has made considerable achievements both in categories and functions. Now, the progress in microelectromechanical systems (MEMS) have resulted in sensors made in a much smaller scale. The smartphone, for example, is composed of a micro-accelerometer, micro gyroscope, gravity sensor, magnetic sensor, light sensor, distance sensor, global position system, etc. Thanks to the smartphone, our living habits, social forms, entertainment, and so on have changed significantly. In the future, with the progress of sensor technology and the gradual maturation of the Internet of Things (IoT), smart cities, artificial intelligence, driverless, virtual reality, etc., the world will change dramatically.

### 1.2. A Bottleneck for Sensors–Power Supply

With the advances of IoT and smart cities, as the signal acquiring fundamental unit, countless sensors will be placed on all manner of locations, and many of them are in inaccessible areas such as long-distance transmission lines, oil and gas pipelines, long-distance optical cables, forests, oceans, etc. In addition, there also has some embedding applications including the implanted medical instruments and the concrete members of important projects, such as bridges, tunnels, huge buildings, substructures of the high-speed rail, etc. In those applications, sensors are looking forward to continuously and self-maintaining work for a long period of time. So, how to power such a large number of sensors is a key problem that needs to be solved. If a cable power supply is used, it is very complex and difficult to set up wires, and a lot of resources are wasted. A common alternative method is using batteries as the power supplier. Even though there are signs of progress in battery technology, limited capacity is an impassable and inherent defect that requires the battery to face unavoidable maintenance and recharge problems. Thus, using a battery as a power source will be unable to fulfill the requirements of the IoT and the smart city; the power supply is a bottleneck of the sensor technology.

### 1.3. The Origin and Development of Self-Powered Sensors and Systems

**Figure 1:**
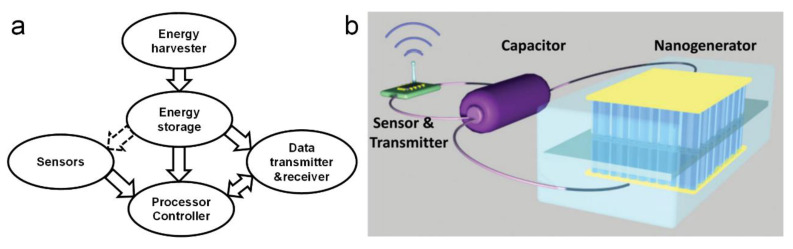
The schematic diagram of the integrated self-powered system. (**a**) An integrated system can be divided into five modules: energy harvester, energy storage, sensors, data processor and controller, and data transmitter and receiver. (**b**) Prototype of an integrated self-powered system using a nanogenerator (NG) as the energy harvester. Reproduced with permission [6]. Copyright 2012, John Wiley & Sons.

To solve the sensor power supply problem, the concept of self-powered sensors and systems was first proposed by Zhong Lin Wang in 2006 [1]. At that time, his group was beginning to create extremely small energy harvesters that can supply electrical power to nanoscale devices. They mainly introduced the piezoelectric nanogenerator (PENG) developed by aligned zinc oxide (ZnO) nanowire arrays, which is a potential technology for converting movement energy, vibration energy, and hydraulic energy into electricity for self-powered nanosystems [2,3,4]. The self-powered nanosystem harvests the ambient energy to power itself, the goal of which is to make it able to operate independently, wirelessly, and sustainably [5]. As shown in Figure 1, a self-powered sensor is constructed of components that are the capability of sensing, communicating, controlling, and responding. Besides the sensing, transmitting, and data processing components, energy harvesting and storage are vital functions of the system [6]. Potential energy sources are solar, thermal, wind, chemical, mechanical, biomass, etc. Using solar cells to harvest solar energy has been used in actual applications [7,8], but a strong dependence on the weather conditions greatly reduces the feasibility of harvesting solar energy as the power supply of sensors. Harvesting electromagnetic (EM) energy has been demonstrated as a power supply of self-powered online monitoring systems for power lines [9]. Although EM energy applies in some specific environment, the feasibility of self-powering through harvesting the ambient energy as the power source was successfully demonstrated. As mechanical energy and fluid energy may exist in many locations in which the sensors will be mounted, they are a good choice for energy harvesting. The function of the nanogenerators (NGs) in energy harvesting has been discussed, the feasibility of which has been demonstrated [10,11,12,13]. Hybrid energy harvesters are also a good choice for sustainable energy harvesting [14,15,16,17,18,19]. In addition, using active sensing technologies to reduce energy demand is also valuable to realize self-powered sensor. Hence, the arrowhead from the energy storage component to the sensors is improved with the dash line in Figure 1a.

The term “self-powered sensor” has a two-fold of meaning. First, it is a sensor that automatically gives out an electric signal when mechanically triggered without an external power source. As we know, most of the sensors used today are passive, which do not give out any signal if there is no power supply. Second, the operation power source provided for the sensor is self-generated. This is possible by considering the active and sleeping mode of a sensor. In many situations, a sensor does not have to send out signal every second; a signal at a certain time interval is enough, such as in environmental monitoring. In such cases, the energy harvested during the “sleep” mode of the sensor can drive it once it is active. This is an approach for its sustainable operation.

Here, we cover the recent progress of self-powered sensors based on NGs in many application fields, including physical sensors, wearable devices, biomedical and health care devices, human–machine interface, chemical and environmental monitoring, smart traffic, smart cities, robotics, and fiber and fabric sensors. We also discuss the challenges and future research directions in the field. We hope this review can bring great benefits to the related research community in the field of self-powering.

## 2. Theory of Nanogenerators

### 2.1. The First Principle Theory of Nanogenerators

The driving force for the NG is Maxwell’s displacement current, which is caused by a time variation of the electric field plus a media polarization term. For power generation, the polarization should contain a term that is contributed by the strain field such as surface contact electrification (CE) (e.g., triboelectric effect) and the piezoelectric effect, which is independent of the presence of an electric field. To explain the contribution made by CE-induced electrostatic charges in Maxwell’s Equations, an additional term ***P_s_*** was added in the displacement vector ***D*** by Wang in 2017 [20]. The reformulated Maxwell’s Equations are

(1) $$\nabla \cdot D^{\prime }=\rho^{\prime }$$

(2) ∇·B=0

(3) $$\nabla \times E=-\frac{\partial B}{\partial t}$$

(4) $$\nabla \times H=J^{\prime }+\frac{\partial D^{\prime }}{\partial t}$$

where $D^{\prime }=\varepsilon_{0}E+P$, and the volume charge density and the density of current density can be redefined as

(5) $$\rho^{\prime }=\rho -\nabla \cdot P_{S}$$

(6) $$J^{\prime }=J+\frac{\partial P_{S}}{\partial t}$$

Then, the newly revised Maxwell’s displacement current can be calculated as

(7) $$J_{D}=\frac{\partial D}{\partial t}=\varepsilon \frac{\partial E}{\partial t}+\frac{\partial P_{S}}{\partial t}$$

**Figure 2:**
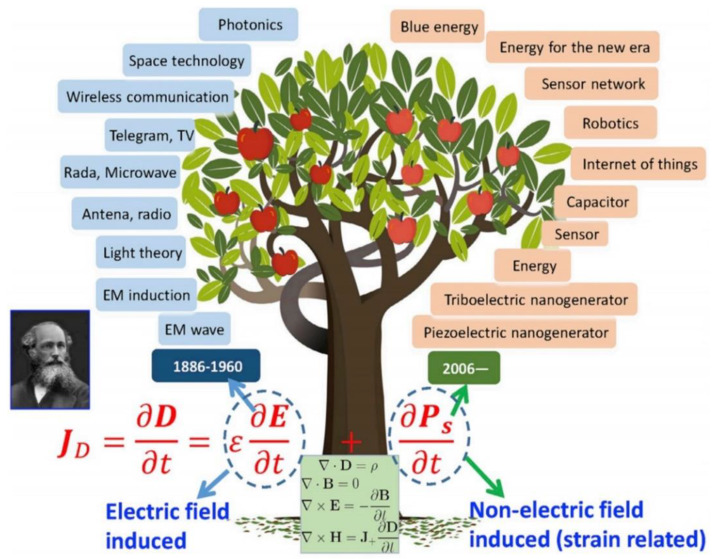
A tree idea to illustrate the newly revised Maxwell’s displacement current: the first term $\varepsilon \frac{\partial E}{\partial t}$ is responsible for the electromagnetic (EM) waves theory; and the newly added term $\frac{\partial P_{S}}{\partial t}$ is the applications of Maxwell’s Equations in energy and sensors, which are the nanogenerators (NGs). Reproduced with permission [21]. Copyright 2020, Elsevier.

Here, the first term $\varepsilon \frac{\partial E}{\partial t}$ represents the displacement current due to time variation electric field and the electric-induced medium polarization, and it can generate the corresponding magnetic field. This is known as the origin of the EM wave, which was first proposed by Maxwell. The second term, $\frac{\partial P_{S}}{\partial t}$, is the displacement current due to nonelectric field but owning to external strain field. This term contributes to the output current of NGs and is related to the driving force (the Wang term). The major fundamental scientific, technological, and practical impacts born from the two components are presented in Figure 2 [21]. The first component of displacement current has driven development of communication and laser technology in the last century. NGs could have extensive applications in IoT, blue energy, and even big data, which will impact the world for the future. NGs could be regarded as another important application of Maxwell’s equations in energy and sensors (after the EM wave theory and technology). For the foreseeable future, the “tree” idea presented in Figure 2 is expected to grow stronger, taller, and larger, which could lead to technological breakthroughs that are expected to impact human society.

### 2.2. Working Principle of Piezoelectric Nanogenerators

**Figure 3:**
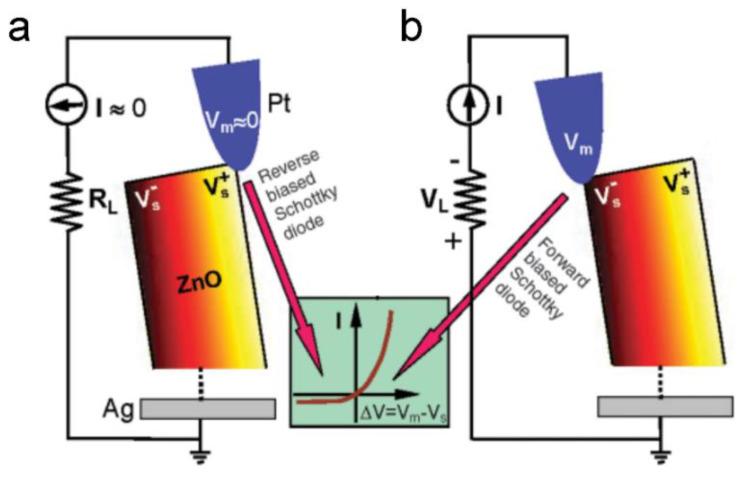
Working principle of a piezoelectric nanogenerator (PENG): contacts between the atomic force microscope (AFM) tip and the semiconductor ZnO nanowire at two reversed local contact potentials (positive and negative), showing reverse and forward biased Schottky rectifying behavior, respectively. This oppositely biased Schottky barrier across the nanowire preserves the piezoelectric charges and later produces the discharge output. The inset shows a typical current–voltage (I–V) relation characteristic of a metal-semiconductor (n-type) Schottky barrier. The process in (**a**) is to separate and maintain the charges as well as build up the potential. The process in (**b**) is to discharge the potential and generates electric current. Reproduced with permission [1]. Copyright 2006, American Association for the Advancement of Science.

Based on the piezoelectric effect, the PENG has the ability to respond to external stimulation and generate an electrical signal. Figure 3 illustrates the working principle of a PENG based on aligned ZnO nanowires (NWs) [1]. The fundamental unit of the PENG is a ZnO NW, the output performance of which is driven and tested by an atomic force microscope (AFM). First, as shown in Figure 3a, the AFM conductive tip that induces the deformation is in contact with the stretched surface of a positive potential $V_{S}^{+}$. The metal tip has a potential of nearly zero, *V_m_* = 0, so the metal tip–ZnO interface is negatively biased for $\Delta V=V_{m}-V_{S}^{+}<0$. Because the assynthesized ZnO NW behave as n-type semiconductors, the metal–ZnO semiconductor (M-S) interface in this case is a reverse-biased Schottky diode, and little current flows across the interface. Second, as shown in Figure 3b, when the AFM tip is in contact with the compressed side of the NW, the M-S interface is positively biased for $V_{L}=V_{m}-V_{S}^{-}>0$. The M-S interface in this case is a positively biased Schottky diode, and it produces a sudden increase in the output electric current. The current is the result of *ΔV*-driven flow of electrons from the semiconductor ZnO NW to the metal tip. The flow of free electrons from the loop through the NW to the tip will neutralize the ionic charges distributed in the volume of the NW and thus will reduce the magnitudes of the potential $V_{S}^{-}$ and $V_{S}^{+}$. Thus, V_L_ starts to drop and reaches zero after all of the ionic charges in the NW are fulyl neutralized. This mechanism explains why the discharge curve in Figure 1a is nearly symmetric. According to the model, the discharge occurs when the NW is bent nearly to its maximum deflection, so V_L_ should have a small offset in reference to the corresponding topography peak along the direction of tip scan. When the NW is deformed by an external stimulating, it will show reverse and forward biased Schottky rectifying behavior, respectively. This oppositely biased Schottky barrier across the nanowire preserves the piezoelectric charges and later produces the discharge output.

### 2.3. Working Principle of Triboelectric Nanogenerators

#### 2.3.1. Triboelectric Nanogenerators

**Figure 4:**
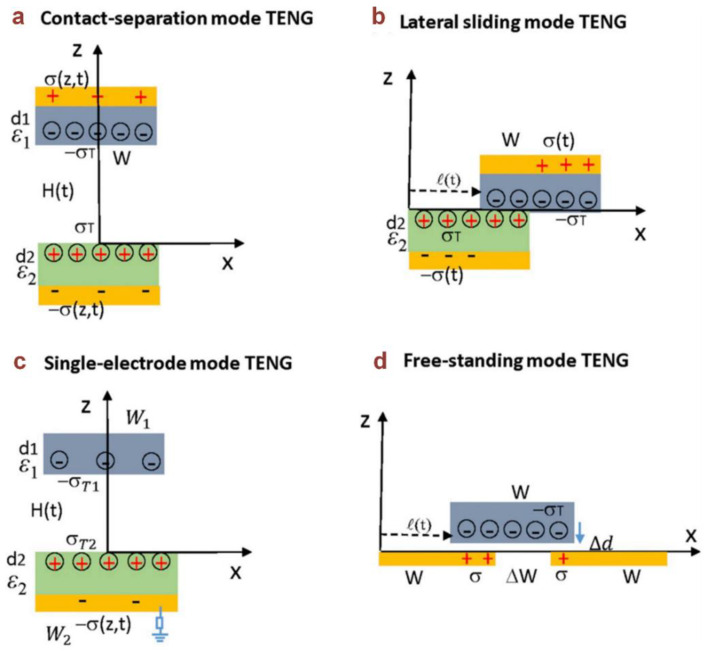
Coordination system and mathematical parameters defined for describing (**a**) contact-separation, (**b**) lateral sliding mode, (**c**) single-electrode mode, and (**d**) free-standing mode triboelectric nanogenerators (TENGs), respectively. Reproduced with permission [21]. Copyright 2020, Elsevier.

A triboelectric nanogenerator (TENG), which is based on the coupling effect of CE and electrostatic induction, was first invented by Wang’s group for harvesting irregular, randomly distributed, and wasted low-frequency mechanical energy and converting it into electric power [22]. It is a field that uses Maxwell’s displacement current as the driving force for effective energy harvesting [22]. Based on a lot of research results, the working modes of TENGs have been summarized as contact-separation mode, lateral sliding mode, single-electrode mode, and free-standing mode, as shown in Figure 4 [21,23]. For the contact-separation mode, when two polymer films contact with and separate from each other, the tribo-charges caused by CE will induce an electric potential difference in the interfacial region and back electrodes, leading to a current flow if there is an external load connected [24]. According to the coordination system and mathematical parameters of the four modes and based on the reformulated Maxwell’s equations (Equations (1)–(4)), the full solutions of the electric potential, output current, and power for a TENG with the four modes have been derived [21].

#### 2.3.2. Mechanisms of CE

**Figure 5:**
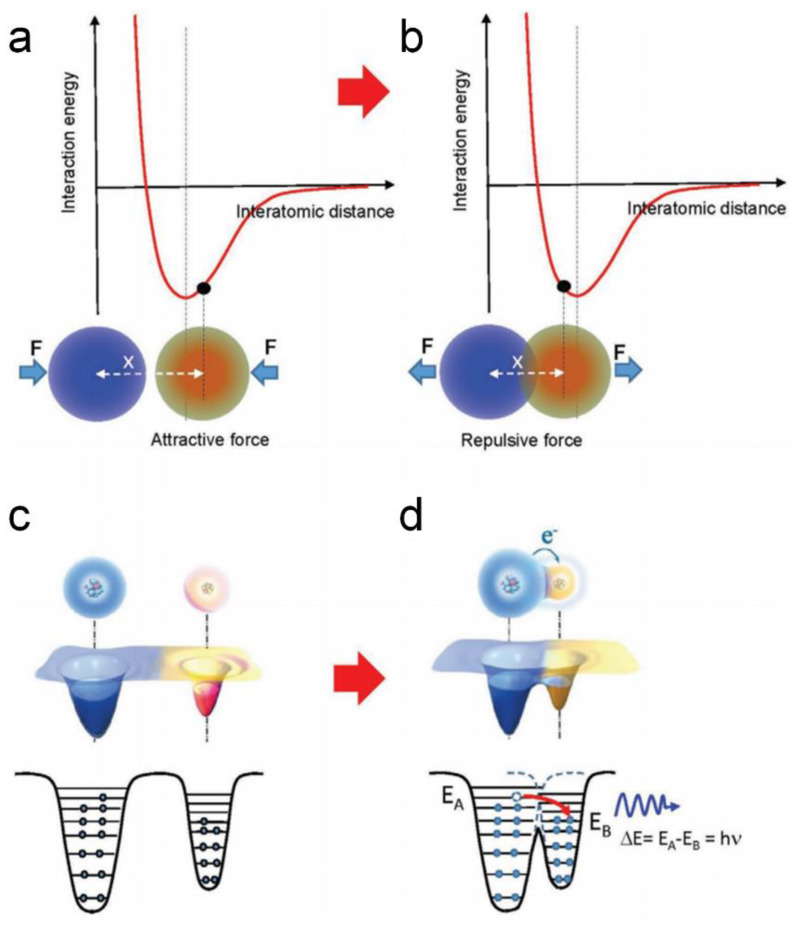
The overlapped electron-cloud model proposed for explaining contact electrification (CE) and charge transfer between two atoms for a general case. (**a**,**b**) Interatomic interaction potential between two atoms when the force between the two is attractive and repulsive, respectively, by applying an external compressive force. Experiments found that electron transfer occurs only when the two atoms are in the repulsive interaction—for example, in the case when the two atoms have strong electron-cloud overlap. (**c**,**d**) Schematic of the electron cloud and potential energy well model of two atoms belonging to two materials A and B when they are separated and in close contact, respectively. Electron transition from atom A to atom B is possible due to the lowered potential barrier by the external force, resulting in the occurrence of CE. This is simply referred to as Wang transition for CE. Reproduced with permission [22]. Copyright 2020, John Wiley & Sons.

CE is a common physical phenomenon between solid–solid, solid–liquid, liquid–liquid, gas–liquid, gas–gas, and gas–solid interactions. The mechanism of CE is systematic discussed in reference [22]. Here, we only retell the mechanism of CE between solid-solid cases. In reference [25], Wang et al. found that CE between two solids is dominated, if not exclusively, by electron transfer. CE between a metal and a dielectric can be well understood using the Fermi level model for metal and the surface states model for a dielectric. CE between a dielectric and a dielectric can be described by the surface states model. It is experimentally found that CE occurs only when the two materials reach a distance shorter than the bonding length, for example, in the repulsive force region in the interaction potential of two atoms (Figure 5b). For a general case, an overlapped electron cloud model is first proposed by Wang et al. to explain the electron transition, in which a strong overlap of the electron cloud between two atoms under stress results in a lowered potential barrier between two atoms, subsequently allowing electron transition from one atom to the other to occur [26]. Mechanical stress is required to make the atoms close enough to maximize the overlapping of electron cloud. This model is considered a generic model for explaining CE between any two materials, and it can be extended to other cases of CE. For simplicity, the electron transition model presented in Figure 5c,d is called the Wang transition for CE. In addition, photon emission is expected during this process, which remains to be experimentally verified.

## 3. Self-Powered Sensors Based on Piezoelectric Nanogenerators

**Figure 6:**
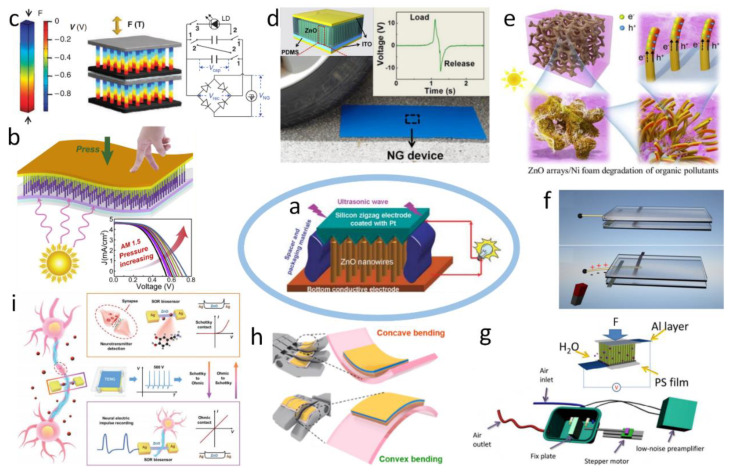
Self-powered sensors based on PENGs. (**a**) Schematic diagram of a PENG. Reproduced with permission [27]. Copyright 2007, American Association for the Advancement of Science. (**b**) Schematic diagram of the as-synthesized flexible solar cell based on n-ZnO/p-SnS core–shell nanowire (NW) array. Reproduced with permission [39]. Copyright 2017, John Wiley & Sons. (**c**) Piezoelectric-nanowire-enabled power source for driving wireless microelectronics. Reproduced with permission [32]. Copyright 2010, Springer Nature. (**d**) Transparent flexible NG as self-powered sensor for transportation monitoring. Reproduced with permission [58]. Copyright 2013, Elsevier. (**e**) Fluid eddy induced piezo-promoted photodegradation of organic dye pollutants in wastewater on ZnO nanorod arrays 3D Ni foam. Reproduced with permission [52]. Copyright 2017, Elsevier. (**f**) Magnetic-force-driven NGs as a noncontact sensor. Reproduced with permission [42]. Copyright 2012, American Chemical Society. (**g**) Enhanced piezo-humidity sensing of Cd-ZnO NWs NG as self-powered/active gas sensor. Reproduced with permission [44]. Copyright 2015, Royal Society of Chemistry. (**h**) Highly sensitive and highly correlative flexible motion sensors based on asymmetric piezotronic effect. Reproduced with permission [57]. Copyright 2018, Elsevier. (**i**) Reversible conversion between Schottky and Ohmic contacts for highly sensitive, multifunctional biosensors. Reproduced with permission [50]. Copyright 2020, John Wiley & Sons.

PENGs produce electricity via strain-induced piezoelectric potential (piezopotential), which is created by inner-crystal ionic polarization. Taking the ZnO NW as the basic unit, Figure 6a is a general structure diagram of a PENG, which is fabricated with vertically aligned ZnO NW [27]. Excepting ZnO NWs, using cadmium sulfide (CdS) [28], cadmium telluride (CdTe) [29], gallium nitride (GaN) [30], NaNbO_3_ [31], and PbZr_x_Ti_1−x_O_3_ (PZT) [32] to make nanobelts [33], nanosheets [34], and nanorods [35], and even two-dimensional MoS_2_ [36] has been demonstrated to fabricated PENGs. PENGs have been successfully applied in energy harvesting [27,32,37,38,39,40,41], sensing [42,43,44,45,46,47], biomedical and healthcare sensors [48,49,50,51], chemical and environmental protection [52,53], fiber and fabric application [54,55,56], robotics [57], and smart cities [58]. In the following, we will briefly introduce some typical applications. Figure 6b shows a flexible solar cell based on n-ZnO/p-SnS core-shell NW array, the relative conversion efficiency of which can be improved to 37.3% through effectively applying the piezo-phototronic effect under a moderate vertical pressure of 320 kPa [39]. The first chemical epitaxial growth of PZT NW arrays at 230 °C and their application as high-output energy converters are shown in Figure 6c [32]. A PENG using a single PZT NWs array provides a current density of 4 µAcm^−2^, a peak output voltage of ~0.7 V, and an average power density of 2.8 mWcm^−3^. This work demonstrates the feasibility of utilizing PENGs to power mobile and even personal microelectronics. Using a flexible polydimethylsiloxane (PDMS) base for the growth of ZnO NWs, packaged transparent flexible PENGs are fabricated, which can be utilized as a self-powered sensor for measuring vehicle weight and monitoring vehicle speed (Figure 6d) [58]. Figure 6e exhibits a nanocomposite with ZnO nanorod arrays vertically growing on 3D nickel (Ni) foam, which is synthesized under hydrothermal conditions and represents both the piezoelectric and photocatalytic functions [52]. Under the role of piezo-promoted photocatalysis, organic pollutants like Rhodamine B (RhB) in wastewater can be degraded. Figure 6f shows a magnetic-force-driven contactless PENG, which is composed of a ZnO nanowire with a magnetic cap sandwiched by two electrodes. Attributed to the introduction of a magnetic cap, the PENG can noncontact harvest mechanical energy and can also be a magnetic sensor [42]. In Figure 6g, a cadmium (Cd) doped ZnO NW PENG as a self-powered gas sensor that exhibits a highly sensitive humidity sensing ability [44]. When exposed to 70% relative humidity, the response of Cd-ZnO (1:10) NWs is up to 85.7, which is much larger than that of undoped ZnO NWs. Cd dopant increases the number of oxygen vacancies in the NWs and results in more adsorption sites on the surface of the NWs. Two flexible motion sensor—ultraviolet-light-emitting diodes based on flexible GaN and PENGs—are described in Figure 6h [57]. Based on asymmetric polarization, they are created across a flexible GaN film. The very high correlations of the sensors enable precise motion measurement. This method measures the magnitude of the strain as well as recognize the direction of bending for realizing multi-functional motion detection sensing devices. Figure 6i provides a conception diagram of a Schottky and Ohmic reversible biosensor for the highly sensitive detection of neurotransmitters and neural electric impulses [50]. The reversible conversion between Schottky contact and Ohmic contact can be achieved by TENG treatment. The Schottky barrier height of the device decreases with the TENG treatment and will gradually recover with time after withdrawing the TENG treatment.

## 4. Self-Powered Sensors Based on Triboelectric Nanogenerators

The following section reviews the recent progress of the TENG in several application fields, including physical sensors, wearable devices, biomedical and healthcare sensors, human–machine interface (HMI), chemical and environmental monitoring, smart traffic, smart homes and smart cities, robotics, and fiber and fabric sensors.

### 4.1. Physical Sensors

#### 4.1.1. Mechanical Motion Sensor

The demand for motion sensors is increasing with the development of intelligent manufacturing, especially for mechanical motion sensors [59]. Current mechanical motion sensors are mainly limited by complex structures, high-costs, and difficulties in fabrication and assembling. Therefore, the mechanical motion sensors based on TENG are widely studied by researchers due to their simple and flexible structures, highly integrated level, and low costs [60]. They can monitor the motion parameters of moving objects and be applied to different research fields [61,62]. The triboelectric sensors for different forms of motion can be divided into three types—position sensors, speed sensors, and acceleration sensors.

**Figure 7:**
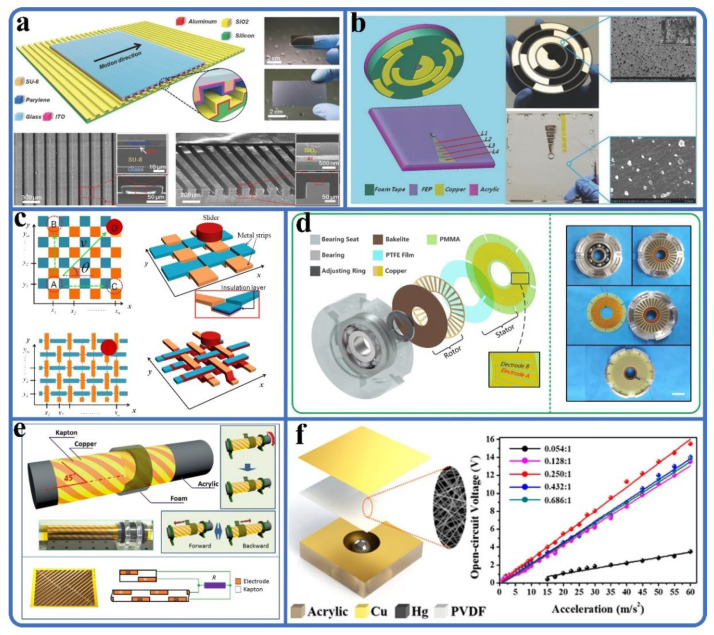
Mechanical motion sensors based on TENG for different motion modes. (**a**) Linear velocity sensor. Reproduced with permission [63]. Copyright 2014, John Wiley & Sons. (**b**) Encoder principle of the angle sensor. Reproduced with permission [64]. Copyright 2015, John Wiley & Sons. (**c**) Motion position monitoring. Reproduced with permission [65]. Copyright 2014, Elsevier. (**d**) Rotation velocity sensor. Reproduced with permission [70]. Copyright 2020, Elsevier. (**e**) Linear-rotary multi-mode speed sensor. Reproduced with permission [66]. Copyright 2014, Elsevier. (**f**) Motion acceleration sensor. Reproduced with permission [71]. Copyright 2017, American Chemical Society.

Motion position monitoring plays a key role in mechanical equipment. The linear position sensors based on TENG are studied. Figure 7a shows a motion sensor based on micro-grated triboelectrification, which has a displacement resolution of 173 nm with a linearity error of 0.02% [63]. In Figure 7b, an angle sensor with four channels coded cooper (Cu) foil material is proposed by Wu et al. [64]. The angle sensor applies the encoder principle to the field of triboelectric sensors and promotes the development of TENG. However, the development of single-dimensional motion sensors is limited because of the limitations of their test motion, so multi-dimensional motion sensors have been rapidly developed. A triboelectric trajectory tracking sensor with a resolution of 250 μm was developed by Han et al. [65], as shown in Figure 7c. Researchers have studied planar multi-dimensional positioning sensors such as a multi-dimensional displacement vector sensor system [66] and a two-dimensional sensor [67]. Also, the researchers studied multi-dimensional sensors with different functions [68,69]. Multi-dimensional sensors have expanded the potential of TENGs in mechanical motion sensing.

Velocity is an extremely important parameter in mechanical motion systems. Researchers have developed a variety of speed sensors based on TENG which have unique applications. In Figure 7d, a triboelectric rotational speed sensor is integrated into the bearing [70], which can be used to measure speeds of 10–1000 rpm with an error below 0.3%. This research has promoted the application of sensors based on TENG in manufacturing. The researchers designed various bearing speed sensors based on TENG [72,73]. The sensor developed by Li et al. can monitor nondestructive damage [74]. Figure 7e shows a dual-mode speed sensor [66] that can detect a linear speed of 0.1–0.6 m/s ± 0.5% and a rotation speed of 300–700 rpm ± 0.9%. In addition, a self-powered motion tracking system designed by Chen et al. can monitor the speed in range ±0.1 m/s, and the acceleration resolution is 0.02 m/s^2^ [75]. There are still many triboelectric rotational motion sensors [66], such as fluid speed sensors [76] and wind speed sensors [77].

Vibration energy exists widely in industrial machinery equipment. The triboelectric acceleration sensors that use vibration energy to realize self-powered and self-sensing can be used to test the acceleration of vibrating objects. An acceleration sensor shown in Figure 7f has a measurement range of 0–60 m/s^2^ with a sensitivity of 0.26 V·s/m^2^ [71]. Moreover, a three-dimensional acceleration sensor [78], a triboelectric acceleration sensor in full space [79], and a multi-axis acceleration sensor with a sensitivity of 3.5 mV·s^−1^ [80] have been studied. In recent years, various acceleration sensors based on TENG have emerged, such as a vibration monitoring sensor with magnetically levitated TENG [81] and a triboelectric accelerometer [82]. It can be seen that triboelectric acceleration sensors can be used as simplified and integrated acceleration sensors.

#### 4.1.2. Fluid Sensors

**Figure 8:**
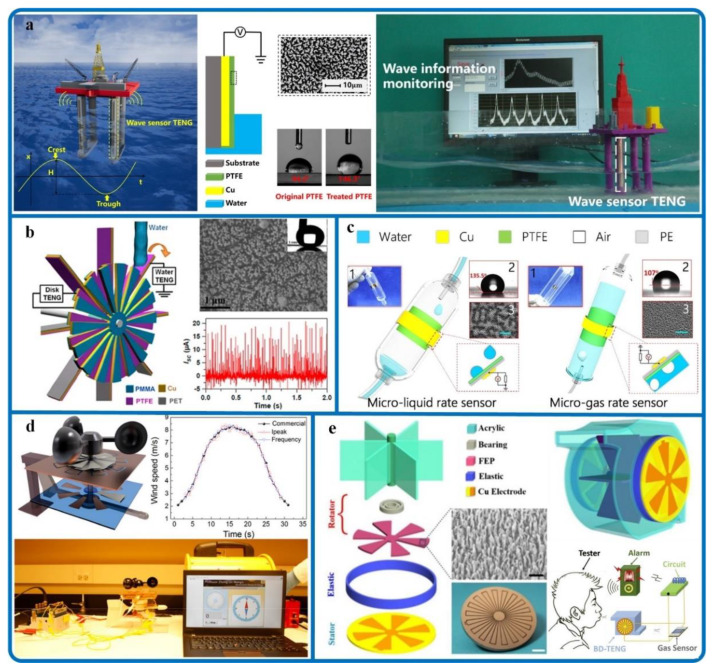
Different forms of fluid sensors based on TENG. (**a**) Schematic of using a water TENG device for self-powered wave sensing based on wave energy. Reproduced with permission [93]. Copyright 2019, Elsevier. (**b**) Schematic of a water TENG device as a self-powered sensor based on water flow energy. Reproduced with permission [95]. Copyright 2014, American Chemical Society. (**c**) The configuration of microliquid flow detection based on droplet energy. Reproduced with permission [96]. Copyright 2016, American Chemical Society. (**d**) Diagram of self-powered wind speed sensor based on wind energy. Reproduced with permission [97]. Copyright 2018, American Chemical Society. (**e**) Device structure of an active alcohol breath analyzer based on gas energy. Reproduced with permission [98]. Copyright 2015, Elsevier.

Fluid sensors are another important part of physical sensors. Most of the mechanical energy available in the environment exists in the form of fluid transmission, such as wave energy [83], water flow energy [84], droplet energy [85,86], wind energy [41,87,88], and gas energy [89,90]. Therefore, much of the research on fluid sensors has been conducted by domestic and overseas scholars. Chengkuo Lee from Singapore proposed a self-powered fishing sensor [91]. Tang et al. proposed a practical bionic jellyfish TENG [92]. The device can wirelessly monitor the fluctuation of the liquid surface accurately. Figure 8a shows a schematic for using a water TENG device for self-powered wave sensing based on wave energy [93]. The wave sensor can detect the wave height in the millimeter range. This research is of great significance for marine engineering construction, marine resource development and utilization, and marine safety. On the other hand, a self-powered water level sensor for ship draft detection with an accuracy of 10 mm was presented by Xu et al. [94].

Fluid energy includes not only large-scale energy sources such as wave energy but also water flow energy, which often presents in many rivers and pipelines [76,99]. Figure 8b exhibits a water wheel hybridized TENG, which can be utilized as a self-powered sensor for perceiving the water flow rate [95]. This hybrid TENG has a good linear proportional relationship between the short-circuit current and the water flow rate. For the identical purpose of water flow harvesting, a rotary TENG is proposed to collect small-scale water energy in our normal living environment (water pipelines, taps, etc.) [100]. Moreover, a rotating TENG designed by Cheng et al. can be used for rust protection, electrostatic scale prevention, and flow sensing by collected water flow energy [94]. Ion concentration in water is a key criterion for evaluating water quality. Excess ions, particles, and bacteria may lead to severe problems in industrial production. For this problem, based on the impedance matching effect of TENG, a self-powered online ion concentration monitor in water transportation is presented [101].

Besides wave energy and water flow energy, droplet energy is also an important source of liquid sensing. Based on droplet energy sensing, a novel self-powered sensor with a super-hydrophobic nanostructured surface is proposed to simultaneously detect the salinity of an aqueous solution [102]. Figure 8c shows a self-powered microfluidic sensor (TMS) to detect liquid and gas flow in real-time using signals generated by droplets/bubbles [96]. Similarly, a microfluidic sensor developed by Lee et al. can simultaneously detect the magnitude and frequency of applied pressure [103]. A single-electrode liquid-solid triboelectric self-powered sensor with a P-type silicon triboelectric layer studied by Zhang et al. can be used for liquid leak detection and identification [104].

Wind/gas energy is another important form of fluid transmission, which is widely used as the medium or driving force of fluid sensors [77,83,105,106]. Based on TENG, a self-powered sensor system is proposed to monitor wind speed and direction simultaneously [97]. A shown in Figure 8d, a flexible contact friction method is used instead of the traditional rigid friction method, which greatly improves sensitivity and reduces the minimum starting wind speed. Based on freestanding woven TENG, a self-powered wind speed sensor is developed to harvest high-altitude wind energy from arbitrary directions [107]. Meanwhile, a TENG based on aeroelastic flutter can be used as an active wind speed sensor [108]. Gas sensing is widely used for monitoring the concentration and species of ambient gases, especially for the detection of toxic or explosive gases. A self-powered triboelectric sensor earlier presented by Guo et al. can detect humidity and airflow rate [109]. A new type of triboelectric-thermoelectric composite generator was designed for gas energy recovery and purification [110]. On the other hand, exhaust gas purification can be performed using the collected exhaust energy. Figure 8e shows a triboelectric sensor driven by blowing air [98]. The detection response time of the sensor is faster than 11 s, and the recovery speed is faster than 20 s. This work is of great significance to promote the development of TENG sensors in the field of gas sensing.

### 4.2. Wearable Devices

Wearable devices are worn directly on the body or integrated into the user’s accessories or clothing, which are portable and are new ways to manage information [111]. They will make a big difference in our lives and perceptions. However, the power supply problem greatly limits its user’s experience. TENG, as new energy technology, brings morning twilight to this problem and offers highly competitive options to acquire user’s information.

#### 4.2.1. Wearable TENGs

**Figure 9:**
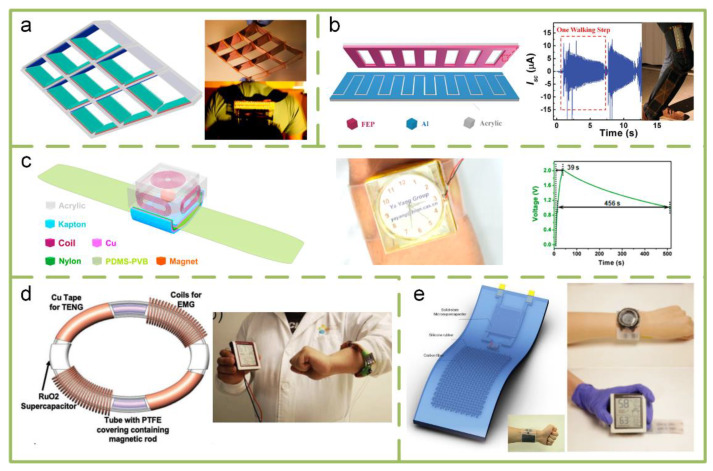
Self-powered sensors based on wearable TENGs. (**a**) A TENG with integrated rhombic gridding for harvesting walking energy. Reproduced with permission [112]. Copyright 2013, American Chemical Society. (**b**) Grating-structured freestanding TENG. Reproduced with permission [30]. Copyright 2014, John Wiley & Sons. (**c**) Hybridized triboelectric-electromagnetic (TE-EM) NG for a self-powered electronic watch. Reproduced with permission [114]. Copyright 2015, American Chemical Society. (**d**) An energy harvesting-storage bracelet. Reproduced with permission [115]. Copyright 2019, John Wiley & Sons. (**e**) MXene electrochemical microsupercapacitor integrated with a TENG as a wearable self-charging power unit. Reproduced with permission [116]. Copyright 2018, Elsevier.

Harvesting the energy in human motion, such as walking, running, hand gesture, etc., as the power source of wearable devices is the most intuitive and feasible technical route. Based on TENG, a variety of structure designs, material selections, and hybrid working principles are implemented for the highly effective harvesting of energy in different motion types. In Figure 9a, a TENG with integrated rhombic gridding is integrate with a backpack to harvest vibration energy from human walking [112]. It is demonstrated as a power source for lighting 40 commercial light-emitting diodes (LEDs). A stack integrated multilayered TENGs was also proposed to harvest vibration energy [79]. With superior synchronization, it outputs a 303 V open-circuit voltage, a 1.14 mA short-circuit current, and a peak power density of 104.6 Wm^−2^. Meanwhile, a grating-structured freestanding TENG is placed on the user’s shank to harvest energy from walking (Figure 9b) [30]. It is shown that each walking step will generate electrical output in a certain period. However, those harvesters have the same disadvantage with a suspended-load backpack reported by Lawrence C. Rome [113]—the large size limits them to only being suitable for certain special uses such as exoskeletons, individual equipment, etc.

Recently, hybrid triboelectric-electromagnetic (TE-EM) energy harvesters have been demonstrated as a good choice for energy harvesting [14,15,16,17,18,19]. The availability of the hybrid design route in wearable devices also has been proven. Figure 9c is a hybrid NG including a TENG and six electromagnetic generators (EMGs). It can effectively harvest biomechanical energy by continuously powering an electronic watch [114]. Through harvesting biomechanical energy during different motion types of the wearer’s wrist, an electronic watch can be continuously powered. The best situation is that it charges a 100 μF capacitor in 39 s, and the stored energy can maintain the watch’s continuous operation for 456 s. Under the inspiration of an EM-based energy harvesting bracelet [117], a hybrid energy harvesting bracelet, which combines a dual EM and TENG, is proposed to harvest wrist motions (Figure 9d) [116]. The bracelet is able to charge a RuO_2_-based microsupercapacitor to 2 V with a single shake of the wrist. Figure 9e shows a watch belt integrated with a TENG composed of carbon fibers and silicone and a MXene electrochemical microsupercapacitor [116]. As a wearable self-charging power source, it can harvest and store the random energy from human activities in a standby mode and provide power to electronics when active. In a word, the common advantage of those studies is that they can be directly integrated into the user’s accessories.

#### 4.2.2. Flexible and Stretchable TENGs

**Figure 10:**
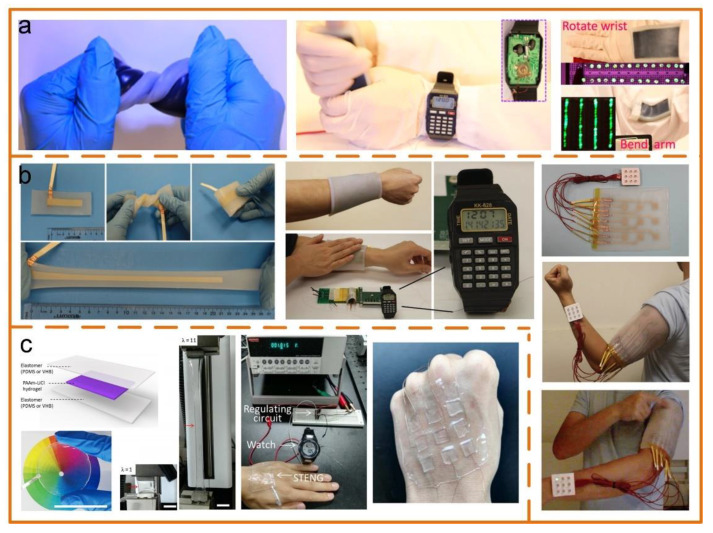
Self-powered sensors based on flexible and stretchable TENGs. (**a**) A stretchable and waterproof TENG based on carbon black electrode. Reproduced with permission [118]. Copyright 2016, American Chemical Society. (**b**) A super-stretchable NG based on Ag NWs electrode. Reproduced with permission [124]. Copyright 2016, John Wiley & Sons. (**c**) Ultrastretchable, transparent TENG based on PAAm-LiCl hydrogel electrode. Reproduced with permission [127]. Copyright 2017, American Association for the Advancement of Science.

Flexible and stretchable TENGs are another design solution to construct self-powered sensors. This TENG usually uses silicone or PDMS to package some electrode materials, such as carbon black, [118,119,120] carbon grease [121], carbon nanotubes (CNTs) [122,123], Ag NWs [124,125,126], PAAm-LiCl hydrogel [127,128,129], liquid metal [85,130,131], etc. Figure 10a is an example of a soft and stretchable TENG based on carbon black [118]. Through combining the stretchable TENG with stretchable supercapacitors, a power system is proposed and used to effectively harvest various kinds of human motion energy, which can drive an electronic watch. The performances of a stretchable TENG composed of PDMS and Ag nanowires are exhibited in Figure 10b [124]. The device shows the capabilities to be twisted and folded in many directions, a uniaxial stretchability of over 300% strain, and various desired deformations. When worn on the forearm, it can continuously power a watch by hand tapping. It can also be used to build an e-skin system for controlling the LEDs by tapping. In those electrode materials, only the PAAm-LiCl hydrogel is transparent, and its average transparency is 98.2%. Based on this, Pu et al. proposed a soft skin-like TENG composed of a hybridizing elastomer and ionic hydrogel, which enables both biomechanical energy harvesting and tactile sensing (Figure 10c) [127]. As an energy harvester, it first achieves an ultrahigh stretchability of 1160% uniaxial strain and a transparency of 96.2% average transmittance under visible light. Through harvesting human motion energy, it can reach an instantaneous peak power of 35 mW m^−2^. Meanwhile, its pressure sensitivity means it can be exploited as an electronic skin for touch/pressure perception.

#### 4.2.3. Smart Shoes

**Figure 11:**
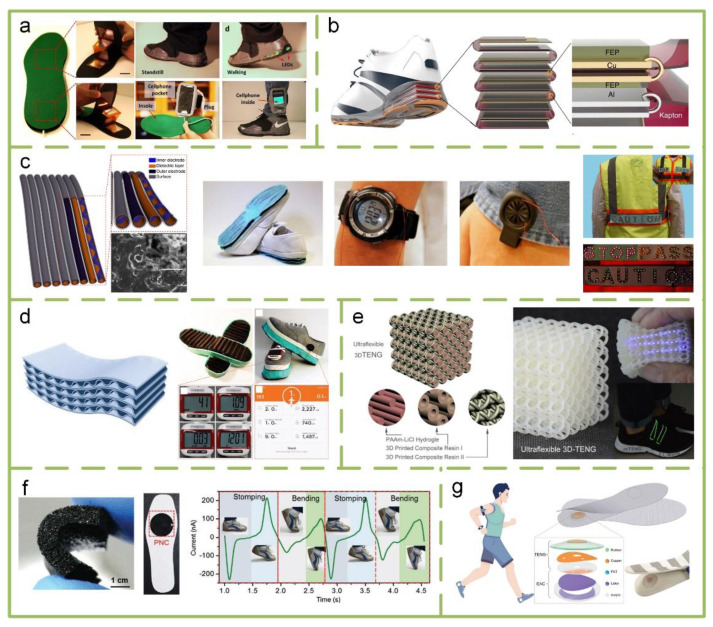
Smart shoes based on TENGs. (**a**) A packaged power-generating insole with built-in flexible multi-layered TENGs. Reproduced with permission [132]. Copyright 2013, Elsevier. (**b**) A multilayer TENG embedded into the sole to exploit human biomechanical energy. Reproduced with permission [133]. Copyright 2015, Springer Nature. (**c**) A soft TENG fabricated by elastomeric materials and a helix inner electrode sticking on a tube with the dielectric layer and outer electrode. Reproduced with permission [119]. Copyright 2016, Springer Nature. (**d**) A TENG with a multilayer elastomeric structure with closely stacked arches as basic functional units as a continuous energy source for wearable electronics. Reproduced with permission [120]. Copyright 2017, John Wiley & Sons. (**e**) A practical, ultraflexible, and three-dimensional TENG fabricated by 3D printing. Reproduced with permission [134]. Copyright 2018, Elsevier. (**f**) Stretchable porous carbon nanotube-elastomer hybrid nanocomposite for harvesting mechanical energy and detecting motion types. Reproduced with permission [135]. Copyright 2017, John Wiley & Sons. (**g**) A TENG-based smart insole for multifunctional gait monitoring. Reproduced with permission [136]. Copyright 2019, John Wiley & Sons.

The feet usually are the most active part of the body during the day. Hence, many researchers study smart shoes based on TENGs, which are generally embedded into insoles or soles. Figure 11a shows a packaged energy harvesting insole embedded with flexible multi-layered TENGs [132]. Through harvesting normal walking energy, the electrical energy generated by the TENGs is stored in a wearable charging gadget for charging portable consumer electronics, such as cellphones. A similar multilayer TENG is embedded into the sole to exploit human walking energy (Figure 11b) [133]. Integrating it with a power management circuit and an energy storage device, a self-powered system can be constructed. With palm tapping as the only energy source, it gives continuous DC electricity with a 1.044 mW output power. Unfortunately, due to the current use of hard materials even with a thin film structure, the lifetime and durability of multilayer TENGs needs to be enhanced. Therefore, using flexible materials to build soft TENGs is a potential design route.

In Figure 11c, Wang et al. proposed a soft TENG fabricated by elastomeric materials and a helix inner electrode adhering to a tube with a dielectric layer and an outer electrode [119]. It has excellent characteristics including stretchability, flexibility, weavability, isotropy, waterproof, and a high surface charge density of 250 µC m^−2^. Another soft TENG with a multilayer elastomeric structure with closely stacked arches as basic functional units is also built in outsoles to harvest energy from walking or jogging (Figure 11d) [120]. Some specific fitness functions are realized on each shoe separately. Three-dimensional printing technology has been introduced to build an ultraflexible 3D TENG, which is composed of ionic hydrogel as the electrode and electrification layer and printed composite resin parts (Figure 11e) [134]. Smart lighting shoes that harvest walking energy to power flexible green LED filaments have been successfully demonstrated. TENGs also can be exploited as active sensors for monitoring motion state. Figure 11f shows a stretchable TENG composed of a PDMS matrix and a CNTs network to harvest mechanical energy [135]. Based on sacrificial templates and a sponge-like structure, the TENG was fabricated by a straightforward two-step method. Driven by external mechanical forces, it generates electricity by the contact electrification between the PDMS matrix and the CNTs network on the surface of inner cavities. The TENG was successfully demonstrated in detecting foot motion types. A TENG with an air-pressure-driven structure assembled into smart insoles also can detect motion types for real-time gait monitoring (Figure 11g) [136].

### 4.3. Biomedical and Healthcare Technology

In biomedical a healthcare technology, according to the installation method, TENGs can be divided into external pasting TENGs and implantable TENGs, which are introduced in the following subsections alongside some smart applications.

#### 4.3.1. External Pasting TENGs

**Figure 12:**
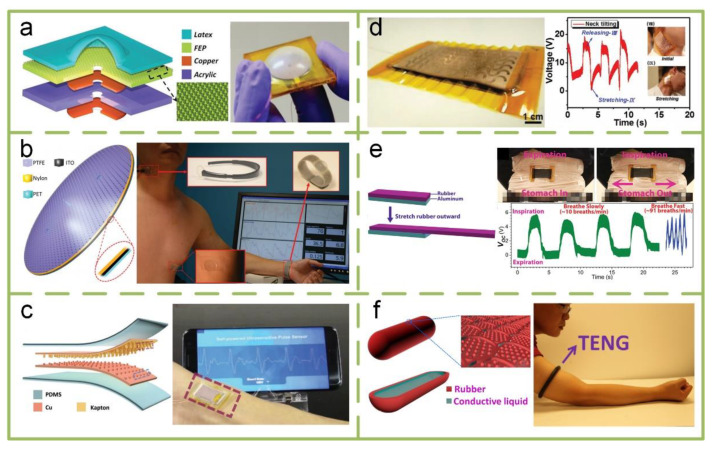
External pasting TENGs for biomedical and healthcare applications. (**a**) Membrane-based self-powered triboelectric sensors for pressure change detection. Reproduced with permission [137]. Copyright 2014, John Wiley & Sons. (**b**) Eardrum-inspired active sensors for self-powered cardiovascular system characterization. Reproduced with permission [138]. Copyright 2015, John Wiley & Sons. (**c**) Self-powered pulse sensor for antidiastole of cardiovascular disease. Reproduced with permission [139]. Copyright 2017, John Wiley & Sons. (**d**) A flexible, stretchable, and shape-adaptive TENG. Reproduced with permission [140]. Copyright 2015, John Wiley & Sons. (**e**) Stretchable-rubber-based TENG and its application as self-powered body motion sensors. Reproduced with permission [141]. Copyright 2015, John Wiley & Sons. (**f**) A highly shape-adaptive, stretchable TENG based on conductive liquid. Reproduced with permission [142]. Copyright 2016, American Association for the Advancement of Science.

The vibrations caused by the pulse and heartbeat can be utilized by TENG, which is an excellent method for biomedical and healthcare purposes. A mechanical structure that can response to vibration is a necessary component in the design of a TENG. Figure 12a is a triboelectric sensor based on a membrane design [137]. It is proposed as a self-powered pressure change sensor, which can generate voltage in response to changes in air pressure. Through integrating it with a signal processing unit as a heartbeat monitor, the open-circuit output voltage reaches about 0.08 V. Figure 12b is another membrane-based sensor, which innovatively couples the contact electrification effect with a structure inspired by a human eardrum [138]. It can provide a superior sensitivity of 51 mV Pa^−1^ with a pressure detection limit down to 2.5 Pa and a fast response time of less than 6 ms. It can be used to measure rapidly changing pressure over a wide range frequency from 0.1 kHz to 3.2 kHz, which enables it to acquire and recover high-frequency throat sound using a single device and to continuously monitor the human low-frequency arterial pulse wave. In addition to membrane-based structures, a square cavity structure has also been designed as a self-powered pulse sensor for antidiastole of cardiovascular disease, as shown in Figure 12c [139]. It has excellent output performance (1.52 V), high peak signal-noise ratio (45 dB), long-term performance (10^7^ cycles), and a low cost.

Potential stretching caused by body movement, such as breath, has also attracted some researchers’ attention. In Figure 12d, a flexible TENG, fabricated by assembling serpentine-patterned electrodes and a wavy-structured Kapton film, can operate at both stretching and compressive modes [140]. When conformably attached onto human skin, it can be utilized as a monitor of the gentle movements of joints and muscles. Another stretchable-rubber-based TENG consisted of a layer of elastic rubber and a layer of aluminum film that acts as the electrode is shown in Figure 12e [141]. It can also be placed on a human body to measure joint motion and breathing. A shape-adaptive TENG fabricated by a conductive liquid contained in a polymer cover is also a good stretchable design (Figure 12f) [142]. It is so flexible that it can be conformed to any curvilinear or three-dimensional surface, and it has been successfully used as a self-powered sensor to monitor biomechanical motion.

#### 4.3.2. Implantable TENGs

**Figure 13:**
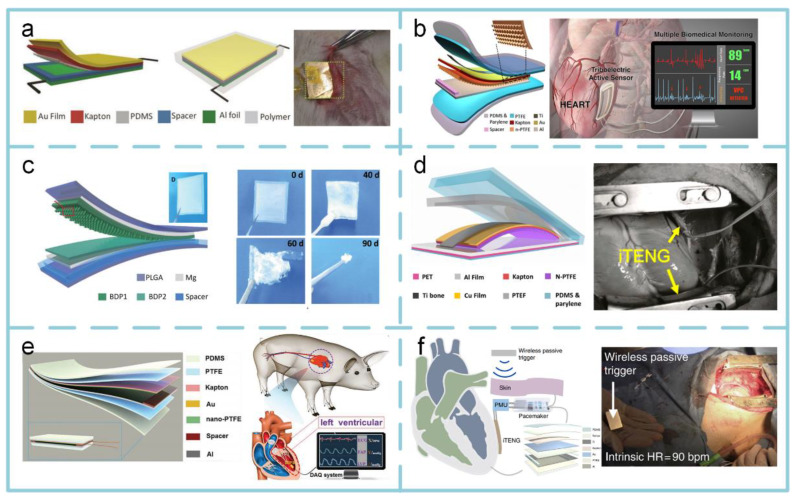
Implantable TENGs for biomedical and healthcare applications. (**a**) In vivo powering of a pacemaker by a breathing-driven implanted TENG. Reproduced with permission [143]. Copyright 2014, John Wiley & Sons. (**b**) A self-powered, one-stop, and multifunctional implantable triboelectric active sensor. Reproduced with permission [144]. Copyright 2016, American Chemical Society. (**c**) A biodegradable TENG as a life-time-designed implantable power source. Reproduced with permission [145]. Copyright 2016, American Association for the Advancement of Science. (**d**) In vivo self-powered wireless cardiac monitoring via an implantable TENG. Reproduced with permission [146]. Copyright 2016, American Chemical Society. (**e**) A transcatheter self-powered ultrasensitive endocardial pressure sensor. Reproduced with permission [147]. Copyright 2019, John Wiley & Sons. (**f**) A symbiotic cardiac pacemaker. Reproduced with permission [148]. Copyright 2019, Springer Nature.

Recently, some researchers have focused on exploiting TENG to harvest heartbeat energy as the power source of a pacemaker. The first work of in vivo biomechanical energy harvesting using a TENG is demonstrated in Figure 13a [143]. The TENG is based on a rectangular cavity designed to harvest heartbeat energy. When attached to a rat’s diaphragm, its typical output current is 0.6 µA. Meanwhile, the rectangular-cavity-based TENG can also be developed as an implantable active sensor for the continuous monitoring of multiple pathological and physiological signs (Figure 13b) [144]. Through testing in human-scale animals, it can monitor heart rates with an accuracy of ~99%. However, due to the limitations of in vivo application, biodegradation is a critical property of this TENG. Figure 13c provides a biodegradable TENG consisting of biodegradable polymers and resorbable metals, which can be degraded and resorbed in an animal body after completing its work cycle without any adverse long-term effects [145]. Its open-circuit voltage and corresponding short-circuit current can reach up to ~40 V and ~1 mA, respectively. Compared with the TENG with a rectangular cavity structure plotted in Figure 13a, when driven by the heartbeat of adult swine, the output voltage and the corresponding current of a TENG with camber cavity structure (Figure 13d) are enhanced by factors of 3.5 and 25, respectively [146]. Changes in endocardial pressure have important clinical significance for heart failure patients with impaired cardiac function. Figure 13e demonstrates that it can be detected by a TENG-based self-powered endocardial pressure sensor, which is integrated with a surgical catheter for minimally invasive implantation [147]. Figure 13f shows a fully implanted symbiotic pacemaker based on an implantable TENG, which realizes energy harvesting and storage as well as cardiac pacing on a large-animal scale [148]. The symbiotic pacemaker successfully corrects sinus arrhythmia and prevents deterioration. It provides an open circuit voltage of 65.2 V, and the energy harvested from each cardiac motion cycle is 0.495 μJ, which is higher than the required endocardial pacing threshold energy (0.377 μJ).

#### 4.3.3. Smart Applications

**Figure 14:**
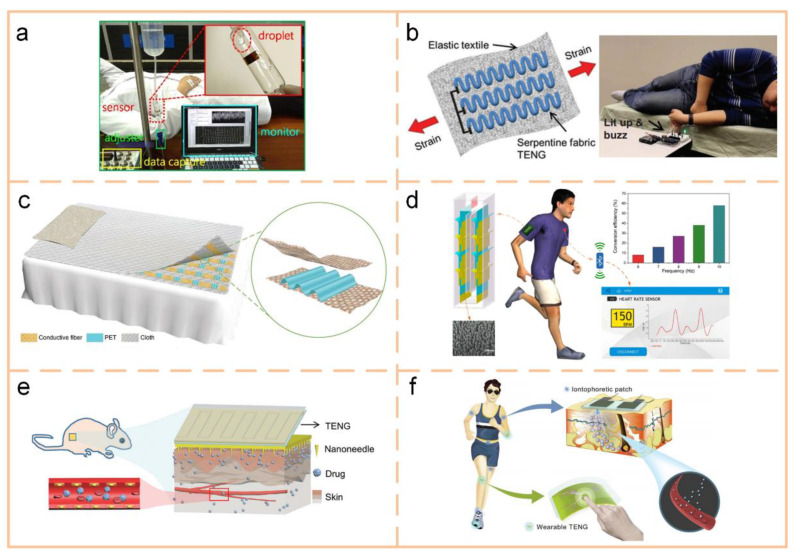
Some smart applications. (**a**) Infusion monitor using a self-powered triboelectric micro liquid flow sensor. Reproduced with permission [95]. Copyright 2016, American Chemical Society. (**b**) Single-thread-based wearable and highly stretchable TENGs and their applications in cloth-based biomedical sensing. Reproduced with permission [149]. Copyright 2013, John Wiley & Sons (**c**) Large-scale and washable smart textiles based on TENG arrays for self-powered sleeping monitoring. Reproduced with permission [150]. Copyright 2018, John Wiley & Sons. (**d**) A TENG-enabled body sensor network for self-powered human heart-rate monitoring. Reproduced with permission [151]. Copyright 2017, American Chemical Society. (**e**) Self-powered intracellular drug delivery by a biomechanical energy-driven TENG. Reproduced with permission [152]. Copyright 2019, John Wiley & Sons. (**f**) A self-powered iontophoretic transdermal drug delivery system driven and regulated by biomechanical motions. Reproduced with permission [153]. Copyright 2020, John Wiley & Sons.

A self-powered triboelectric microfluidic sensor can be a fluid sensor, as discussed in Figure 8c, but can also be used to build an infusion monitor, as shown in Figure 14a [95]. In Figure 14b, a triboelectric thread using a silicone rubber-coated stainless-steel thread can be stitched into a bedspread, which can be used to construct a smart bed to wirelessly monitor human motion [149]. When the occupant moves to the edge of bed, the triboelectric thread will be active, triggering the transmitter to wirelessly transmit a warning signal to the receiver. Then, it will buzz and power up an LED to alert the user. Another sleeping monitoring application is exhibited in Figure 14c; it is a pressure-sensitive, washable, and large-scale smart textile [150]. Figure 14d shows a self-powered wireless body sensor network system for heart-rate monitoring [151]. The system is integrated with a downy-structure-based TENG, a heart-rate sensor, a power management circuit, a signal processing unit, and a Bluetooth. By harvesting human walking energy, the TENG is capable of immediately and sustainably driving the system. Transdermal drug delivery (TDD) systems with feedback control have attracted wide research and clinical interest owing to their individual advantages of self-administration, convenience, and safety. In Figure 14e, through simple finger friction or hand slapping of the wearable TENGs, a transdermal biomolecule delivery with an over threefold depth enhancement is successfully realized in mice [152]. In Figure 14f, a wearable TENG is utilized as an energy harvester and motion sensor to transform biomechanical motions into electricity for iontophoresis without additional power sources, while a hydrogel-based soft patch with side-by-side electrodes is designed to enable noninvasive iontophoretic TDD [153].

### 4.4. Human–Machine Interface

**Figure 15:**
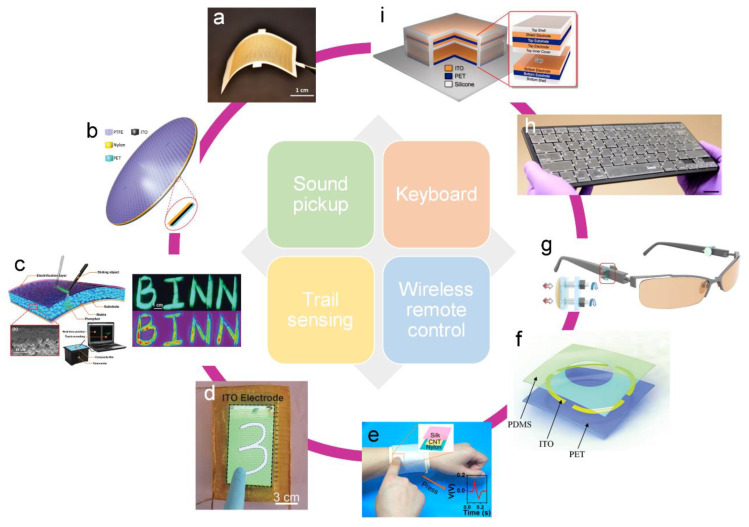
TENGs for human–machine interface applications. (**a**) A paper-based TENG for self-powered sound recording. Reproduced with permission [154]. Copyright 2015, American Chemical Society. (**b**) Eardrum-inspired active sensors for self-powered throat-attached anti-interference voice recognition. Reproduced with permission [138]. Copyright 2015, John Wiley & Sons. (**c**) Dynamic triboelectrification-induced electroluminescence and its use in visualized sensing. Reproduced with permission [155]. Copyright 2016, John Wiley & Sons. (**d**) Self-powered high-resolution and pressure-sensitive triboelectric sensor matrix for real-time tactile mapping. Reproduced with permission [156]. Copyright 2016, John Wiley & Sons. (**e**) Screen-printed washable electronic textiles as self-powered touch gesture tribo-sensors. Reproduced with permission [157]. Copyright 2018, American Chemical Society. (**f**) Self-powered noncontact electronic skin for motion sensing. Reproduced with permission [158]. Copyright 2018, John Wiley & Sons. (**g**) Eye-motion-triggered self-powered mechnosensational communication system using TENG. Reproduced with permission [159]. Copyright 2017, American Association for the Advancement of Science. (**h**) Personalized keystroke dynamics for self-powered human–machine interfacing. Reproduced with permission [98]. Copyright 2015, American Chemical Society. (**i**) Keystroke-dynamics-enabled authentication and identification using TENG array. Reproduced with permission [160]. Copyright 2018, Elsevier.

Human-machine interface research uses various techniques to design and realize interfaces between users and machines, where machines are defined as any mechanical or electrical devices that assist in human tasks [24]. Here, we will discuss TENG-enabled HMI applications including sound pickup, trail sensing, wireless remote controls, and keyboards. In Figure 15a, a rollable, 125 μm thickness, paper-based TENG has been utilized as a sound pickup to scavenge soundwave energy [154]. Based on membranes with rationally designed holes on one side, it works in the contact-separation mode. Owing to the superior advantages of its thin structure, flexibility, and broad working bandwidth, a self-powered microphone with rolled structure is demonstrated for all-sound recording without an angular dependence. An eardrum-inspired active sensor for use as a pulse and heartbeat monitoring sensor is represented in Figure 12b, which can be used in self-powered throat-attached anti-interference voice recognition (Figure 15b) [138]. It can pick up and recover a human voice even in a windy or extremely noisy environment. Figure 15c shows a dynamic triboelectrification-induced electroluminescence (TIEL) fabricated by a multilayered composite material that is used in visualized sensing [155]. The composite consists of a substrate, an electrification layer made of fluorinated ethylene propylene, and a luminescent layer composed of poly(methyl methacrylate) matrix and zinc sulfide: Cu phosphor particles. When a separate object slides against the electrification layer, transient light emission from the luminescent layer can be observed along the motion trajectory. Based on a self-powered pressure-sensitive and high-resolution TENG matrix, real-time tactile mapping can be realized, as demonstrated in Figure 15d [156]. A flexible 16 × 16 pixelated TENG matrix shows a resolution of 5 dpi. It can map single-point and multi-point tactile stimuli in real time through the multichannel data acquisition method. It also has long-term durability and an excellent pressure sensitivity of 0.06 kPa^−1^. A wireless remote control application based on a washable electronic textile (WET) is shown in Figure 15e [157]. The WET is constructed of three layers—the top silk fabric layer, the middle CNT electrode array layer, and the bottom nylon fabric layer. The CNT ink is printed on nylon fabric to construct the electrode array layer, and the polyurethane added in the CNT ink is used to realize the washability of the electrode. As a touch sensor incorporated into a wristband, the WET can detect the touch of human fingertips and output a pulse signal. With the cooperation of some electronic modules, it can enable wirelessly trigger home appliances. Figure 15f represents a self-powered noncontact electronic skin capable of perceiving the motion of a surface electrified object across the plane parallel to that of the electronic skin based on electrostatic induction and triboelectric effects [158]. It can serve as a human–machine interface due to its ability to sense noncontact motions and has been demonstrated by the electronic-skin-controlled Tetris game platform. Considering the mechanical micromotion of the skin around the corners of the eyes as a trigger signal source, mechnosensational TENGs mounted on a pair of ordinary glasses can be exploited as a method to help people suffering from “locked-in syndrome” to communicate with the world, as shown in Figure 15g [159]. The computer keyboard, as the most common, accessible, effective, and reliable device used for human–machine interfacing and information exchange, has undoubtedly attracted some research into application development based on TENG to analyze human behavior via keystrokes. Figure 15h shows a self-powered, TENG-based intelligent keyboard (IKB), which can response to the mechanical stimuli applied to the keyboard and convert it into electronic signals [98]. During the typing process, through detecting both the dynamic time intervals between and during the inputting of letters and the force used for each typing action, the IKB has the potential to be used as a smart security system. Moreover, the IKB is able to identify personal characteristics from different individuals, assisted by the behavioral biometric of keystroke dynamics. Another keyboard with a TENG array shown in Figure 15i is also a strong proof of the keystroke dynamic functions. A two-factor, pressure-enhanced keystroke-dynamics-based security system is capable of authenticating and even identifying users through their unique typing behavior [160]. The promising application of this novel system in the computing and financial industry can push cyber security to a much higher level, where leaked passwords would possibly be of no concern.

### 4.5. Chemical and Environmental Monitoring

**Figure 16:**
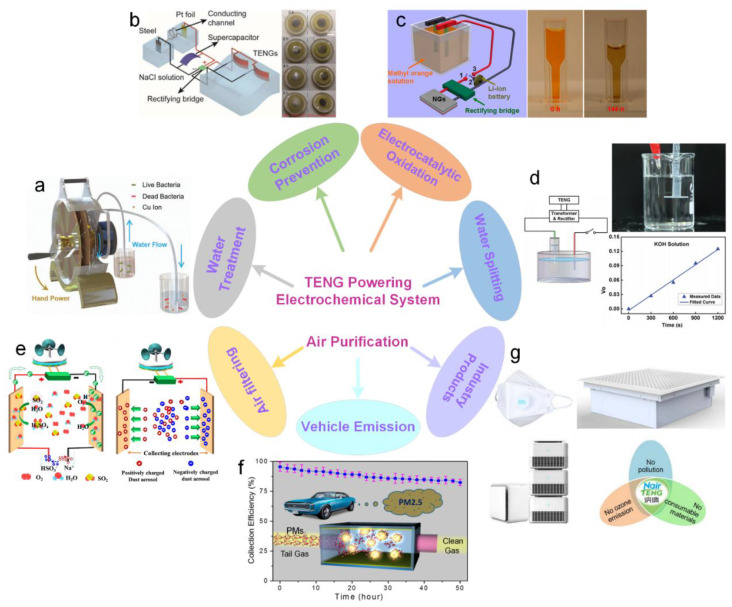
TENGs powering electrochemical systems and used for air purification. (**a**) TriboPump: a low-cost, hand-powered water disinfection system. Reproduced with permission [161]. Copyright 2019, John Wiley & Sons. (**b**) A self-powered system based on TENGs and supercapacitors for metal corrosion prevention. Reproduced with permission [166]. Copyright 2015, Royal Society of Chemistry. (**c**) A hybrid energy cell for the degradation of methyl orange by self-powered electrocatalytic oxidation. Reproduced with permission [168]. Copyright 2013, American Chemical Society. (**d**) Self-powered water splitting using flowing kinetic energy. Reproduced with permission [171]. Copyright 2015, John Wiley & Sons. (**e**) Self-powered cleaning of air pollution by wind-driven TENG. Reproduced with permission [172]. Copyright 2015, Elsevier. (**f**) Removal of particulate matter emissions from a vehicle using a self-powered triboelectric filter. Reproduced with permission [173]. Copyright 2015, American Chemical Society. (**g**) Industry products developed based on the triboelectric air filtering technology. Reproduced with permission [174]. Copyright 2019, IOP Publishing.

With the development of TENG, some researchers have studied its use in electrochemical systems. A TENG generally acts as a power source for electrochemical systems, which can be categorized into water treatment [161,162,163,164,165], corrosion prevention [166,167], electrocatalytic oxidation [168,169], and water splitting [170,171]. Figure 16a gives a waste water treatment system, called TriboPump, which is composed of three functional units—a disk TENG as the power source, a tubular coaxial-electrode copper ionization cell (CECIC) as the disinfection device, and a coaxial mechanical structure including a water pump [161]. With the integration of CECIC and TENG, the system can effectively disinfect water while pumping it solely by hand power. As presented in Figure 16b, Li et al. designed a network of TENGs and supercapacitors to harvest water energy for preventing metal corrosion [166]. The corrosion results indicate that the system can prevent about 80% of corrosion for Q235 steel in 0.5 M NaCl solution. Figure 16c demonstrates that Ta ENG can also be used to drive an electrocatalytic oxidation system. A hybrid energy cell consisting of a TENG and a PZT film-based pyroelectric nanogenerator (PNG) was developed to power a self-powered electrocatalytic process [168]. The cells can simultaneously or individually harvest mechanical and thermal energies, which ensures that the degradation of methyl orange (MO) can be implemented without using an external power source. The mechanical energy harvested by the TENG is saved in a Li-ion battery and then drives the degradation of MO by electrocatalytic oxidation. The PNG directly powers the degradation of MO, where the degradation percentage is up to 80% after 144 h. The water splitting application based on TENG is shown in Figure 16d, in which a TENG and a water splitting unit are integrated [171]. The hydrogen production rate in 30% (w.t.) potassium hydroxide (KOH) solution reaches 6.25 × 10^−3^ mL·min^−1^ with the TNEG’s spinning speed of 600 rpm. Particularly, when the KOH solution is replaced by pure water, the hydrogen generation efficiency of the system is four times than that of an electrochemical workstation at 10 V.

With the rapid development of society, air pollution has become more and more serious, and the haze caused by particulate matters (PMs) has become a global problem. Hence, action is urgently needed to seek effective technologies to remove airborne PMs or other pollutants. TENG has been successfully developed to realize efficient air filtering [110,172,175,176,177] and vehicle emission treatment [173,178]. In fact, TENG-based air purification products are already available in the market [174]. Figure 16e is an example of TENG- based air filtering, which is driven by harvesting natural wind energy [172]. This self-powered air filtering system not only adsorbs dust particles in air but also oxidizes SO_2_ without producing byproducts. Therefore, it may have the potential to improve the haze situation. PM pollution from vehicle exhaust has become one of the main pollution sources in urban environments. Although diesel particulate filters have been applied in heavy diesel vehicles, there is no particulate filter for most light-duty vehicles or gasoline cars due to the limitation of high price. According to the triboelectrification effect, a self-powered TENG filter is introduced to remove PMs from vehicle exhaust (Figure 16f) [173]. Through installing the triboelectic filter on a vehicle to harvest the self-vibration of the tailpipe, it shows a mass collection efficiency of ~95.5% for PM2.5. The NairTENG Company is developing industry products based on the TENG air cleaning technology, including a face mask, a distributed household triboelectric air purifier, and an embedded triboelectric purification system, (Figure 16g) [174]. No pollution, no ozone emission, and no consumable materials are the unique characteristics of these industry products.

**Figure 17:**
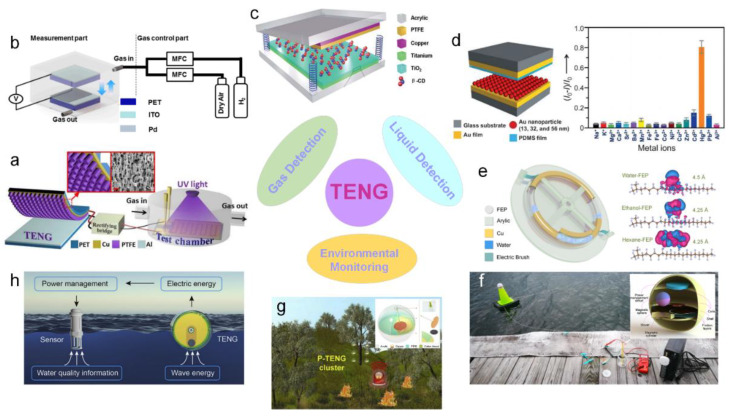
TENGs for gas and liquid detection and environmental monitoring. (**a**) Self-powered room temperature NO_2_ detection driven by TENG under ultraviolet (UV) illumination. Reproduced with permission [179]. Copyright 2018, Elsevier. (**b**). A triboelectric hydrogen gas sensor with a Pd functionalized surface. Reproduced with permission [180]. Copyright 2016, MDPI. (**c**) β-cyclodextrin-enhanced triboelectrification for self-powered phenol detection and electrochemical degradation. Reproduced with permission [181]. Copyright 2015, Royal Society of Chemistry. (**d**) A self-powered triboelectric nanosensor for mercury ion detection. Reproduced with permission [182]. Copyright 2013, John Wiley & Sons. (**e**) Direct-current rotary-tubular TENGs based on liquid-dielectrics contact chemical composition analysis. Reproduced with permission [183]. Copyright 2019, American Chemical Society. (**f**) A hybridized triboelectric–electromagnetic water wave energy harvester based on a magnetic sphere for environmental monitoring. Reproduced with permission [19]. Copyright 2019, American Chemical Society. (**g**) Super-robust and frequency-multiplied TENG harvesting wind energy for a self-powered wireless forest fire warning system. Reproduced with permission [184]. Copyright 2019, Elsevier. (**h**) High-performance TENGs for self-powered and real-time water quality mapping. Reproduced with permission [185]. Copyright 2019, Elsevier.

Figure 17 exhibits the applications of TENGs for gas and liquid detection and environmental monitoring. First, TENG can be utilized as a power source of some gas sensors. In Figure 17a, a TENG is used to power a chemoresistive gas sensor based on zinc oxide-reduced graphene oxide (ZnO-RGO) hybrid films to detect the concentration of NO_2_ [179]. The self-powered triboelectric gas sensor composed of ZnO-RGO composite films exhibit superior response (~16.8), sensitivity, and selectivity to that of a pure ZnO film. Moreover, the TENG also can be directly developed as an active gas sensor. By coating palladium (Pd) on a indium tin oxides (ITO) player, a TENG composed of an ITO layer and polyethylene terephthalate (PET) film can be utilized as an H_2_ sensor, as shown in Figure 17b [180]. When it is exposed to H_2_, its output voltage is directly proportional to the H_2_ concentration. This is attributed to the changes of the work function of the Pd-coated surface, which alters triboelectric charging behavior. Reproducible and sensitive sensor response was observed for up 1% H_2_ exposure. This approach can easily be adopted to develop triboelectric gas sensors detecting other gas species. Through a surface modified by β-cyclodextrin to enhance triboelectrification, a TENG can be used as an self-powered phenol sensor, as shown in Figure 17c [181]. Its detection sensitivity reaches 0.01 µM^−1^ in the sensing range of 10 µM to 100 µM. Meanwhile, as shown in Figure 17d, an Au nanoparticles (AuNPs)-based TENG is developed as a selective and highly sensitive nanosensor for Hg^2+^ ions detection [182]. The metal layer of the TENG is covered by the assembly of AuNPs, which is a further modification of 3-mercaptopropionic acid molecules. The function of Hg^2+^ ions detection is attributed to the different triboelectric polarity of AuNPs and Hg^2+^ ions. Under optimum conditions, this nanosensor has a detection limit of 30 nM and a linear range from 100 nM to 5 µM. Using Cu electrodes around a fluorinated ethylene propylene tube and placing two electric brushes bilaterally anchored, a liquid−dielectrics interface–based TENG is proposed in Figure 17e [183]. The electric brushes are introduced to convert the AC output current into DC output current, which enables energy harvesting without rectification. This TENG design has also been demonstrated for chemical detection in liquid composition and moisture content analysis.

Harvesting wave, wind, and solar energies as a power source for environmental sensors for the detection of temperature, water quality, humility, oxygen level, pH, etc. is an effective and promising method to realize self-sustaining and maintenance-free environmental monitoring. Figure 17f shows a self-powered wireless water temperature alarm system fabricated by a hybridized TE-EM water wave energy harvester (WWEH) based on a magnetic sphere, a power management circuit, a temperature switch, and an alarm device [19]. The WWEH consistently harvests the wave energy and converts it into electric energy to store in a supercapacitor. When the environmental temperature reaches beyond the threshold value, the temperature switch will be closed to promote the WWEH-driven alarm device. This application has confirmed the technical feasibility of the WWEH-based self-powered wireless sensor networks for environmental monitoring. Figure 17g shows a self-powered wireless forest fire warning system based on a pendulum-inspired TENG that harvests wind energy [184]. The pendulum structure not only boosts the output frequency but also significantly enhances the energy harvesting efficiency through transforming impact kinetic energy into potential energy. A self-powered water quality monitoring system based on a high-performance tandem disk TENG is demonstrated in Figure 17h [185]. Due to its radial grating structure design, this TENG can be effectively stimulated by slow water waves, boosting its average power to 7.5 mW.

### 4.6. Smart Traffic

**Figure 18:**
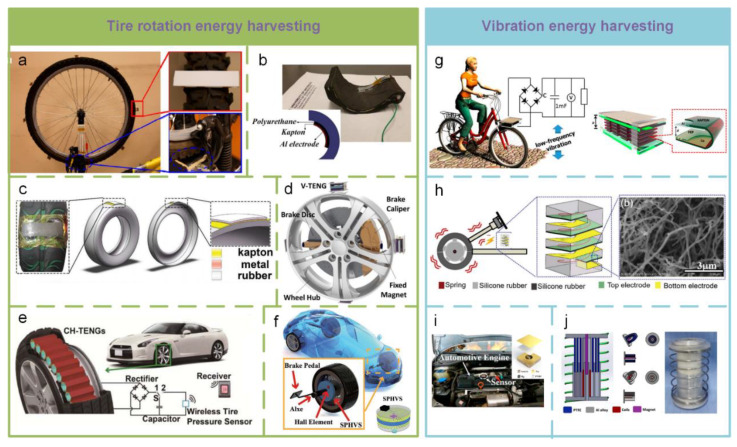
TENGs for tire rotation energy and vehicle’s vibration energy harvesting. (**a**) A single-electrode-based rotating TENG for harvesting energy from tires. Reproduced with permission [186]. Copyright 2014, American Chemical Society. (**b**) A triboelectric self-powered sensor for tire condition monitoring. Reproduced with permission [187]. Copyright 2017, John Wiley & Sons. (**c**) A TENG for boosting smart green tires. Reproduced with permission [188]. Copyright 2019, John Wiley & Sons. (**d**) An on-vehicle TENG-enabled self-powered sensor for tire pressure monitoring. Reproduced with permission [179]. Copyright 2018, Elsevier. (**e**) Compressible hexagonal-structured TENGs for harvesting tire rotation energy. Reproduced with permission [189]. Copyright 2018, Elsevier. (**f**) Self-powered hall vehicle sensors based on TENGs. Reproduced with permission [190]. Copyright 2018, John Wiley & Sons. (**g**) Harvesting ambient vibration energy over a wide frequency range for self-powered electronics. Reproduced with permission [191]. Copyright 2017, American Chemical Society. (**h**) A soft and robust spring-based TENG for harvesting arbitrary directional vibration energy and self-powered vibration sensing. Reproduced with permission [192]. Copyright 2018, John Wiley & Sons. (**i**) A self-powered acceleration sensor based on a liquid metal TENG for vibration monitoring. Reproduced with permission [85]. Copyright 2014, American Chemical Society. (**j**) A spring-assisted hybrid TE-EM NG for harvesting low-frequency vibration. Reproduced with permission [193]. Copyright 2018, Royal Society of Chemistry.

Harvesting tire rotation energy is the best way to power a tire pressure monitoring system (TPMS) and other smart applications. Figure 18a shows a single-electrode-based TENG composed of a rotary acrylic disc with polytetrafluoroethylene (PTFE) blades and an aluminum (Al) electrode fixed on the base [186]. The design of this TENG successfully mitigates the need for a connection structure between the rotational and fixed parts for harvesting rotational energy from a rotating tire. However, the PTFE blades attached around the tire reduce the usefulness of this design. In Figure 18b, a TENG made of flexible polymeric materials is directly attached on the inside of a tire to monitor tire conditions [187]. The experimental results demonstrate that the proposed sensor has the potential to detect tire pressure and force. The application of this sensor for TPMSs is a significant step in the development of smart tires. Wu et al. (Figure 18c) [188] proposed green energy tires composed of TENGs and silica tread rubber. The TENGs harvest the frictional energy and convert it into electricity without changing the process of traditional tire production, which can be utilized to power the electronics measuring tire pressure and automobile safety. The silica-based tread compound sharply cuts off the rolling resistance, provides all-around traction without compromising braking, improves fuel efficiency, and reduces CO_2_ emissions. The on-vehicle magnetic TENG shown in Figure 18d is also designed to scavenge tire rotating energy and serve as a direct power source for a pressure sensor [179]. TENG has also been used to power a commercial wireless sensor for transmitting real-time temperature data to a receiver. For enhancing the energy harvesting effect, an array of compressible hexagonal-structured TENGs is proposed and shown in Figure 18e [189]. The TENGs can be connected in parallel and fixed to the inside surface of a rubber tire. When a TENG is connected with eight units and works under a speed of 2.51 m/s and a weight load of 10 N, the maximum output power reaches 1.9 mW. During 30-day continuous testing, the proposed TENG shows stable and durable features and can drive a wireless tire pressure sensor working once every 14 min. This work demonstrates the feasibility of the TENG as a power source of TPMS. Growing demand in vehicle safety systems increases the requirement that electronic devices be self-powered, durable, and stable. In Figure 18f, a self-powered Hall vehicle sensor is proposed for vehicle safety system, which consists of a Hall element, a magnet, a TENG, and a power management circuit [190]. This work clearly provides a promising solution for braking monitoring and speed sensing and indicates the potential applications of power sources for the self-powered electronic devices in the vehicle industry.

The vibration energy contained in the running process of vehicles is another alternative energy source. In Figure 18g, an elastic TENG with the similar multilayer design shown in Figure 11b is fabricated to harvest the low-frequency vibration energy [191]. When the vibration frequency is as low as 7 Hz, the proposed TENG can provide a maximum output power density of 102 W·m^−3^. And a stable output current can be maintained from 5 Hz to 25 Hz. Through harvesting the vibration energy from a bicycle, it can sustainably power a speedometer. In Figure 18h, a robust and soft TENG composed of a silicone rubber-spring helical structure with nanocomposite-based elastomeric electrodes is proposed to place on the vehicle for harvesting vibration energy [192]. It can serve as a self-powered vibration sensor. The highly sensitive and self-powered acceleration sensor represented in Figure 7f also has potential applications in equipment vibration monitoring and troubleshooting, which is demonstrated by fixing it onto an automotive engine, as shown in Figure 18i [85]. A spring-assisted hybrid NG composed of an EMG and a TENG is proposed to harvest low-frequency vibration energy (Figure 18j). With the operating frequency is as low as 2 Hz, the TENG and EMG can produce a peak power of 1682 µW with a resistive load of 50 MΩ and about 57.6 mW with a resistive load of 2000 Ω, respectively.

**Figure 19:**
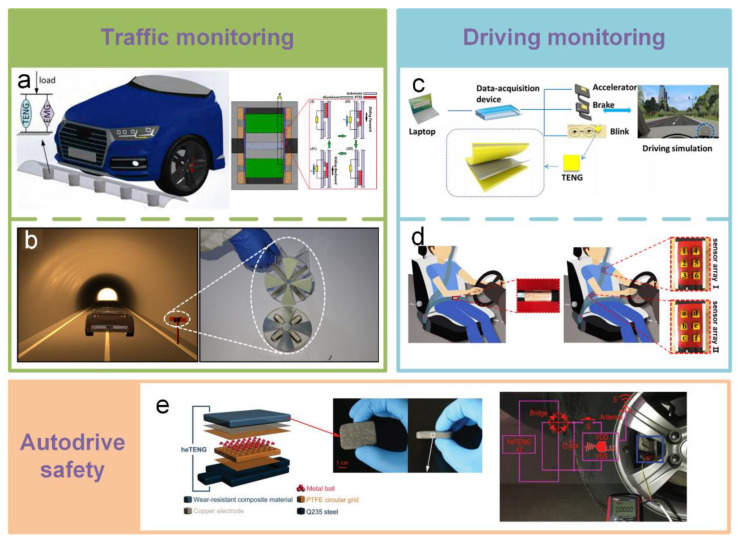
TENGs for smart traffic applications based on TENG. (**a**) A hybridized TE-EM self-powered sensor for traffic monitoring. Reproduced with permission [194]. Copyright 2017, Elsevier. (**b**) Rotating-disk-based hybridized TE-EM NG for sustainably powering wireless traffic volume sensors. Reproduced with permission [195]. Copyright 2016, American Chemical Society. (**c**) TENG as a highly sensitive self-powered sensor for driver behavior monitoring. Reproduced with permission [158]. Copyright 2018, Elsevier. (**d**) A self-powered smart safety belt enabled by TENGs for driving status monitoring. Reproduced with permission [199]. Copyright 2019, Elsevier. (**e**) Harsh-environmental-resistant TENG and its applications in Autodrive safety warning. Reproduced with permission [192]. Copyright 2018, John Wiley & Sons.

Based on the excellent performance of TENG, some smart applications have been implemented, such as traffic monitoring [194,195,196,197,198], driving monitoring [158,199,200], Autodrive safety [192], etc. Figure 19a, presents a hybridized TE-EM generator composed of four freestanding TENGs and four EMGs developed as a self-powered sensor for road traffic monitoring [194]. With the combination of TENG and EMG, it enables energy generation even with very low frequencies and small displacements. Due to its strong output performance and high sensitivity, this system has the potential for sensing applications and traffic monitoring and can harvest energy to boost a self-powered monitoring system. Another hybrid NG based on a rotate disk structure acts as a sustainable power source for a self-powered active wireless traffic volume sensor (Figure 19b) [195]. It can effectively harvest energy from wind generated by a moving vehicle through a tunnel. Then, the output power will trigger a counter through a wireless transmitter for real-time monitoring of the traffic volume in the tunnel. Driver behavior status monitoring is an important route for traffic safety. The driving behavioral parameters are usually acquired by sensitive but expensive sensors, such as an electroencephalograph, eye tracker, etc. Some researchers have tried to usage TENG to acquire driving behavioral parameters. In Figure 19c, a flexible, robust, and pressure-sensitive TENG fabricated by Kapton materials and Al foil acts as an effective sensing device for self-powered driver behavior monitoring [158]. The TENGs are attached to the accelerator, brake, and even around the driver’s eyes to collect the driving behavioral parameters for the analysis of driving behaviors. Furthermore, an auxetic polyurethane TENG and an arch-shaped TENG are developed to construct a self-powered smart safety belt to monitor the turning and forward position actions of the driver (Figure 19d) [199]. The auxetic polyurethane TENG is integrated into the horizontal strip of the safety belt to monitor the driver’s forward position, which is related with aggressive and abrupt deceleration. Two arch-shaped TENGs are attached on the waist and shoulder locations of the safety belt’s diagonal strip. They are used to measure the turning angles and directions of the driver, respectively. The application of TENG in Autodrive safety is described in Figure 17e [192]. Based on the design of dual working modes (single electrode mode and freestanding mode), a harsh environmental TENG (heTENG) is fabricated to enable both the harvesting of sliding/vibration energy and self-powered vibrational sensing. By employing micro–nanocomposite for triboelectric materials, high temperature resistance, wear resistance, and high hardness are achieved in a TENG. The heTENG has the potential to be directly used as a key supporting component, such as vehicle’s brake pads. Moreover, the output performances of the heTENG reach 221 V, 27.9 µA·cm^−2^, and 33.4 µC·cm^−2^. In addition, due to its vibration sensitivity, the heTENG is successfully used to build a vehicle’s self-powered smart braking system and a sensor network, which can automatically produce accurate early warning signals, such as an indicator for tire overloading and pressure and a reminder for brake replacement.

### 4.7. Smart City Applications

**Figure 20:**
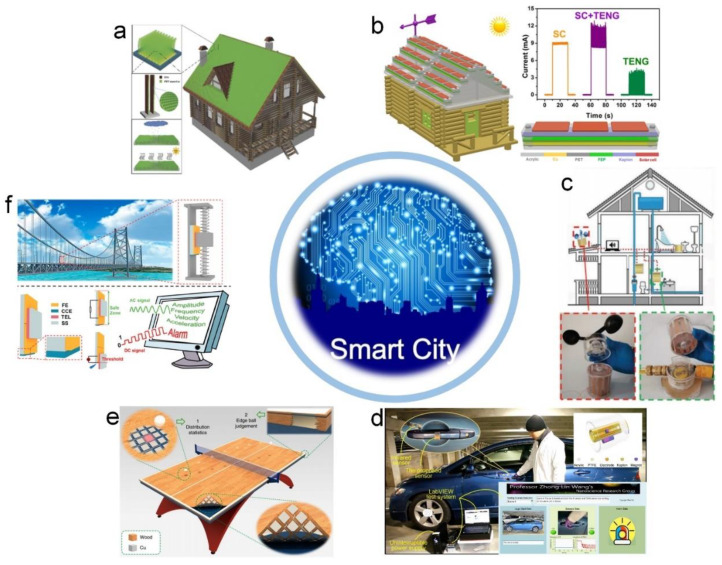
TENGs for smart city applications. (**a**) Lawn-structured TENGs for scavenging sweeping wind energy on rooftops. Reproduced with permission [201]. Copyright 2016, John Wiley & Sons. (**b**) Efficient scavenging of solar and wind energies in a smart city. Reproduced with permission [202]. Copyright 2016, American Chemical Society. (**c**) An easily assembled electromagnetic-triboelectric hybrid NG driven by magnetic coupling for fluid energy harvesting and self-powered flow monitoring in a smart home/city. Reproduced with permission [203]. Copyright 2019, John Wiley & Sons. (**d**) A multifunctional sensor based on translational-rotary TENG for vehicle safety applications. Reproduced with permission [204]. Copyright 2019, John Wiley & Sons. (**e**) Flexible and durable wood-based TENGs for self-powered sensing in athletic big data analytics. Reproduced with permission [205]. Copyright 2019, Springer Nature. (**f**) A fully self-powered vibration monitoring system driven by dual-mode TENGs. Reproduced with permission [206]. Copyright 2020, American Chemical Society.

Some smart city applications based on TENGs are exhibited in Figure 20. Figure 20a presents a laminar TENG array fabricated using vertically free-standing polymer strips composed of ITO-coated PET thin film to harvest natural wind energy at any blowing direction [201]. This array has a kelp forest morphology, resulting in the TENGs working on the contact-separation mode. When the wind passes through the array, every strip may sway independently, driving them to contact and separate from each other. An array consisting of 60 strips was fabricated and installed onto a rooftop to harvest the natural wind energy, and the generated power density of it reaches 2.37 Wm^−2^. Taking a 300 m^2^ rooftop area of a common house as an example, it may be covered with 10 arrays, and the corresponding output power is expected to be 7.11 kW, which is a sustainable power source for home electronics. Another rooftop-based energy harvesting application is a hybridized NG composed of a solar cell and a TENG, as shown in Figure 20b [202]. When installing on the tops of city buildings or public facilities, it can individually or simultaneously harvest solar and wind energies. With the same device area of about 22 mm × 120 mm, the output power delivered by the solar cell and the TENG can reach about 8 mW and 26 mW, respectively. Zhong et al. studied methods for harvesting the water flow energy from our living environment to conveniently and efficiently power small household electric devices [203]. A hybrid generator consisting of a rotary EMG and a TENG with the freestanding mode is proposed to be conveniently integrated with a water turbine and a wind cup for harvesting fluid energy. The energy generated by it is sufficient for some household electronic devices, such as a temperature sensor and a humidity sensor. These results demonstrate that TENG has the potential to improve the power supplying and sensing technologies in smart city.

Based on a translational-rotary magnetic mechanism, a cylindrical TENG is proposed as a self-powered multifunctional sensor (MS) to measure force, acceleration, and rotational parameters [204]. The MS can easily give a response to even a weak strike, so it has also been utilized to build a vehicle safety application, as shown in Figure 20d. The MS is placed on the handle of a car door to perceive the action of the handle. To further enhance the efficiency of this application, an infrared sensor is fixed near the handle to distinguish the presence of the hand. If a hand tries to pull the handle of a locked car, an alarm will sound. The feasibility of the TENGs for smart sport monitoring is shown in Figure 20e [206]. Through integrating durable and flexible wood-based TENGs into a ping-pong table, a smart ping-pong table can be built that can act as the foundation to construct a self-powered edge ball judgement system and a self-powered falling point distribution statistical system. These systems can provide real-time competition assistance and training guidance for both referees and athletes. This work can expand the application area of TENG to smart sport monitoring and assisting and can also boost the development of big data in the intelligent sports industry. In the application area of structural health monitoring, a TENG can act as a self-powered vibration sensor [82,206]. In Figure 20f, based on a dual-mode TENG, a self-powered vibration sensor is proposed to realize the continuous and real-time perception of vibration characteristics [206]. During the vibration-safe range, the TENG with AC output can continuously acquire the vibration characteristics and drive the signal transmitter. More critically, once the vibration amplitude overshoots the danger threshold, the TENG will immediately convert the AC output into DC output, which will directly trigger the alarm system to precisely forecast danger.

### 4.8. Robotics

**Figure 21:**
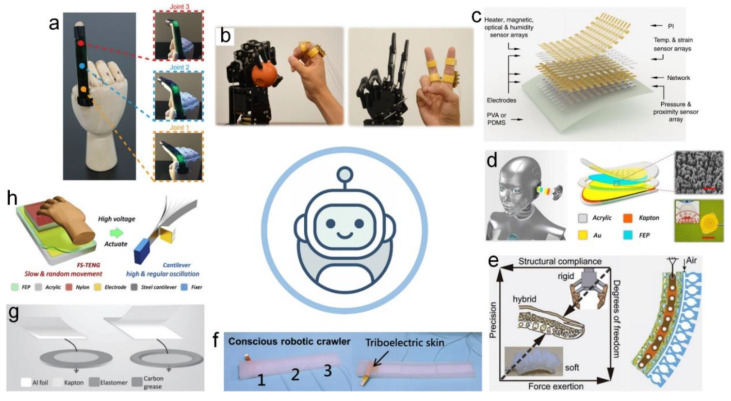
TENGs for robotics applications. (**a**) Stretchable triboelectric–photonic smart skin for tactile and gesture sensing. Reproduced with permission [207]. Copyright 2018, John Wiley & Sons. (**b**) Rotation sensing and gesture control of a robot joint via triboelectric quantization sensor. Reproduced with permission [208]. Copyright 2018, Elsevier. (**c**) Skin-inspired highly stretchable and conformable matrix networks for multifunctional sensing. Reproduced with permission [177]. Copyright 2018, Springer Nature. (**d**) A highly sensitive, self-powered triboelectric auditory sensor for social robotics and hearing aids. Reproduced with permission [209]. Copyright 2018, American Association for the Advancement of Science. (**e**) A TENG as a self-powered sensor for a soft-rigid hybrid actuator. Reproduced with permission [210]. Copyright 2019, John Wiley & Sons. (**f**) Actively perceiving and responsive soft robots enabled by self-powered, highly extensible, and highly sensitive triboelectric proximity and pressure-sensing skins. Reproduced with permission [211]. Copyright 2018, John Wiley & Sons. (**g**) Stimulating acrylic elastomers by a TENG for self-powered electronic skin and artificial muscle. Reproduced with permission [212]. Copyright 2016, John Wiley & Sons. (**h**) An actuation and sensor-integrated self-powered cantilever system based on TENG technology. Reproduced with permission [88]. Copyright 2019, Elsevier.

The applications of TENGs in robotics are focused on sensing and high voltage driving. The stretchable triboelectric-photonic smart skin (STPS) shown in Figure 21a enables multidimensional tactile and gesture sensing for a robotic hand via integration as a conformal covering [207]. It can detect external touch and respond to different gestures with joints bending. Based on a TENG with grating-sliding structure, another joint motion application as a robotic hand synchronous control system is shown in Figure 21b [208]. The grating-sliding design ensures a TENG with an ultrahigh sensitivity to mechanical displacement. It can therefore be directly used to quantify a joint’s flexion-extension degree/speed. The minimum resolution angle of the fabricated TENG is 3.8°, which can be further enhanced via reducing the grating width. Figure 21c presents a skin-inspired conformable and highly stretchable matrix network composed of a heater, magnetic sensor arrays, optical and humidity sensor arrays, temperature and strain sensor arrays, and pressure and proximity sensor arrays [177]. By integrating these multifunctional sensor arrays into an e-skin, its sensing ability has been expanded (but not limited) to in-plane strain, pressure, proximity, magnetic field, temperature, light, and humidity. A personalized intelligent prosthesis was fabricated and has proven its usage in real-time temperature estimation and spatial pressure mapping. Some other signal acquiring approaches of robotics are focused on the auditory system and the visual system. Based on a membrane-based TENG, a self-powered auditory sensor (TAS) is proposed to build an electronic auditory system for an external hearing aid in intelligent robotic applications (Figure 21d) [209]. The TAS shows an ultrahigh sensitivity of 110 millivolts/decibel with a broadband response from 100 Hz to 5000 Hz. When incorporated with intelligent robotic devices, it presents accurate voice recognition and high-quality music recording for accomplishing intelligent human–robot interaction. A conductive sponge/porous silicone-based TENG (SS-TENG) is proposed as a tactile sensor for a hybrid actuator (Figure 21e) [210]. Based on the SS-TENGs, a soft–rigid hybrid actuator can be formed to construct a bionic skeleton–muscle–skin hybrid gripper. The gripper can catch different objects and give feedback via electric signals, which has potential applications in bionic robots. In Figure 21f, a tribo-skin via self-generating electricity presents the ability to response the external stimuli, such as proximity, contact, and pressure [211]. Through integrating the tribo-skin to some soft actuators, it promotes soft robots that have the ability to implement various actively perceiving and interactive tasks, such as actively sensing their working states, muscle motions, textile dampness, and even subtle human physiological signals. The high output performance of TENGs can power electrochemical system and even drive some robotics. In this area, dielectric elastomers (actuator materials) present excellent performance as artificial muscles, are limited by high driving voltage, are the preferred target, and have been demonstrated in Figure 21g [212]. In this application, a self-powered actuation system is constructed using a contact-separation mode TENG as the high voltage power source to directly drive a thin film dielectric elastomer actuator (DEA). When the system works stably within the contact–separation velocity of 0.1–10 cm·s^−1^, a TENG with a tribo surface area of 100 cm^2^ can drive an expansion strain of 14.5% for the DEA with an electrode diameter of 0.6 cm. In Figure 21h, the high output voltage of a freestanding mode TENG is used to trigger a one-end fixed steel cantilever beam (MC) with an integrated real-time oscillation-monitoring sensor (ROS) through a trigger electrode (TE). When the output voltage of the TENG is applied between the MC and TE, the MC can be driven to keep a stable oscillation. In this process, the low-frequency mechanical motion of the TENG (5–200 mm/s) can be converted into the high-frequency oscillation of the MC (up to 100 Hz). Compared with the AC actuation method, the DC trigger strategy is universal to any metal cantilever systems. Moreover, the integrated ROS presents a sensitivity of 223 mV/mm, which is about three times higher than that of a commercial laser micro-motion meter. This work provides us with a new method for using the TENG-triggered cantilever as the driving structure to construct robotics.

### 4.9. Fiber and Fabric Sensors

**Figure 22:**
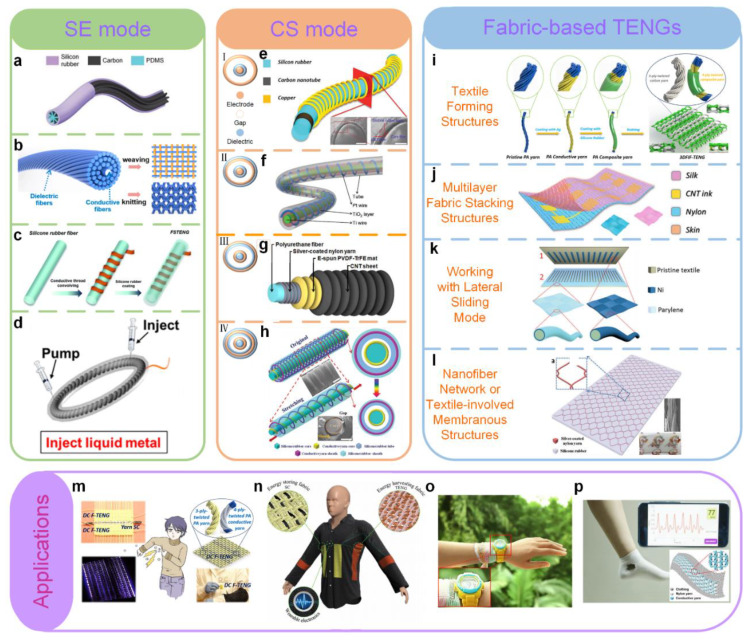
Fiber and fabric-based TENGs and some smart applications. (**a**) Typical design of dielectric polymers directly wrapped around core fiber electrodes to build SE mode fiber-based TENGs. Reproduced with permission [214]. Copyright 2018, John Wiley & Sons. (**b**) Typical design of tightly twining artificial fibers around a conductive core fiber to build SE mode fiber-based TENGs. Reproduced with permission [215]. Copyright 2017, American Chemical Society. (**c**) Typical design of twining fiber electrodes on the surface of stretchable fibers to build SE mode fiber-based TENGs. Reproduced with permission [216]. Copyright 2017, Royal Society of Chemistry. (**d**) Typical design of injecting nontoxic liquid metals into stretchable tubes to build SE mode fiber-based TENGs. Reproduced with permission [217]. Copyright 2014, American Chemical Society. (**e**) Typical design of type I (dielectric layer wrapped around inner electrode as the core and only outer electrode as the shell). Reproduced with permission [218]. Copyright 2017, John Wiley & Sons. (**f**) Typical design of type II (dielectric layer wrapped around an inner electrode as the core and the same configuration as the shell). Reproduced with permission [219]. Copyright 2016, John Wiley & Sons. (**g**) Typical design of type III (inner electrode wound on a dielectric fiber as the core and the same configuration as the shell). Reproduced with permission [220]. Copyright 2016, Springer Nature. (**h**) Typical design of type IV (the outer surface of the type III further covered by a dielectric or encapsulation layer). Reproduced with permission [221]. Copyright 2018, John Wiley & Sons. (**i**) Typical design of fabric-based TENGs with textile forming structures. Reproduced with permission [222]. Copyright 2020, Elsevier. (**j**) Typical design of fabric-based TENGs with multilayer fabric stacking structures. Reproduced with permission [157]. Copyright 2018, American Chemical Society. (**k**) Typical design of fabric-based TENGs working with lateral sliding mode. Reproduced with permission [219]. Copyright 2016, John Wiley & Sons. (**l**) Typical design of fabric-based TENGs with nanofiber reticular or textile-involved membranous structures. Reproduced with permission [223]. Copyright 2018, John Wiley & Sons. (**m**) Direct current fabric TENG for bio-motion energy harvesting. Reproduced with permission [224]. Copyright 2020, American Chemical Society. (**n**) Scheme of a self-charging power textile. It integrates the supercapacitor yarns as energy-storing fabrics, the TENG cloth as energy-harvesting fabrics, and wearable electronics. Reproduced with permission [225]. Copyright 2016, John Wiley & Sons. (**o**) A micro-cable structured textile for simultaneously harvesting solar and mechanical energy to continuous powering an electronic watch in a wearable manner. Reproduced with permission [226]. Copyright 2016, Springer Nature. (**p**) A machine-knitted washable sensor array textile for precise epidermal physiological signal monitoring. Reproduced with permission [227]. Copyright 2020, American Association for the Advancement of Science.

Introducing TENG technology into conventional textiles injects new vitality into the development of smart textiles. Textile-based TENGs are highly desirable for human-oriented self-powered sensors and next-generation wearable energy harvesters that are expected to be long-lasting, lightweight, breathable, washable, and deformable. Fiber and fabric TENGs have been systematic generalized and detailed by Dong et al. (2020) [55]. Fiber-based TENGs (FiTENGs) can be directly used to build self-powered sensors and are the smallest design unit to construct fabric-based TENGs (FaTENGs) through weaving or knitting. In the design of single FiTENGs, the most common design structures are coaxial and core–shell configurations. According to the difference in working modes, they can be further categorized as single-electrode (SE) mode and contact–separation (CS) mode. The common design of the single-fiber-based TENGs with SE mode (SE-TENGs) is composed of an inner fiber electrode and an outer dielectric layer cladding around the electrode to protect it. Meanwhile, the single-fiber-based TENGs with CS mode (CS-TENGs) are usually designed as a core-shell structure to provide a gap between them, which is the critical feature to ensure the contact-separation process. The simplest structure of SE-TENGs is directly wrapping the dielectric polymers around core fiber electrodes [149]. In addition to using metal wire and even silver-coated fiber, the cotton threads coated with multiwalled carbon nanotubes using a homemade CNT ink via a “dipping and drying” method also can be used as the fiber electrode [213]. However, by introducing the space between the electrode and the dielectric polymers, the contact-separation process can also exist in the SE mode. As shown in Figure 22a, a FiTENG consists of a timbo-like inner PDMS core coated with a carbon electrode and an outer silicon tube [214]. Due to the existing timo-like shape, the FiTENG can act as a force and bend sensor. As textile-based TENGs will have potential contact with skin, artificial commercial fibers are gradually used to replace polymers to avoid itching. In Figure 22b, through tightly twining artificial fibers around a conductive core fiber, a coaxial fiber as a SE-TENG is fabricated and can be further knitted or woven into fabrics to realize the large-scale production [215]. In Figure 22c, stretchiness is achieved by twining fiber electrodes around stretchable fibers [216]. In Figure 22d, a super stretchable SE-TENG is achieved via injecting nontoxic liquid metals, e.g., EGaIn and Galinstan, into stretchable silicone rubber tube [217]. However, those designs are only suitable for sensing. In order to realize large-scale stretchiness, choosing a suitable weaving process is much more effective.

Although SE-TENG is easy to fabricate and use, low and unstable output blocks its extensive application. To enhance the output power and stability of FiTENGs, the CS mode is a better choice. According to the difference in structures, CS-TENGs are categorized into the following four main types [55]: a dielectric layer wrapped around an inner electrode as the core and only an outer electrode as the shell (type I, Figure 22I), a dielectric layer wrapped around an inner electrode as the core and the same configuration as the shell (type II, Figure 22II), an inner electrode wound on a dielectric fiber as the core and the same configuration as the shell (type III, Figure 22III), and the outer surface of the type III further covered by a dielectric or encapsulation layer (type IV, Figure 22IV). Figure 11e–h presents some typical fiber-based TENGs corresponding to four types, respectively. No matter what type is chosen, one of the basic design criteria is that the selection of triboelectric materials must ensure the difference between the outer side of the inner core column and the inner side of the outer shell tube. Moreover, a suitable fabricating process must be specified to ensure the existence of the gap. However, the disadvantages of the complicated structure design of the CS mode and the difficult fabricating process of the gap lead the CS-TENGs not being suitable for large-scale production. So, the potential applications of the CS-TENGs are limited.

In reference [55], according to structural characteristics, fabrication methods, and operational modes, FaTENGs can be classified into the following four main types: FaTENGs with textile forming structures, FaTENGs with multilayer fabric stacking structures, FaTENGs working with lateral sliding mode, and FaTENGs with nanofiber reticular or textile-involved membranous structures. The typical examples corresponding to these types are exhibited in Figure 16i–l, respectively. Large-scale industrialized production of FaTENGs is one of basic premises for their wide commercial application. However, it is disappointing to note that most FaTENGs are manually fabricated. Only a few successful studies have aimed large-scale industrialized production, and these only focus on the FaTENGs with textile-forming structures [215,228]. In order to realize the production of smart clothes, the most valuable and promising design uses FaTENGs with textile-forming structures. Therefore, research should be focused on the design of suitable fiber materials and weaving structures in order to get the system to market as quickly as possible. The performance of textile-based TENGs with plain- and 2/1 twill-woven textile-forming structures has been tested [229]. Compared with the plain-woven textile structure, textile-based TENGs with 2/1 twill-woven textile structure exhibit higher output voltages.

Here, we will give some interesting applications based on textile-based TENGs. In Figure 22m, a DC FaTENG with the most common plain structure is proposed to harvest biomotion energy via the annoying and harmful electrostatic breakdown phenomenon of clothes [224]. A sample with a size of 1.5 cm × 3.5 cm can easily light 416 serially connected LEDs. Moreover, some supercapacitor yarns also can be woven into it as an energy storage unit to power some electronic devices, such as a calculator and a hygrothermograph, which shows high efficiency and great convenience in practice. In Figure 22n, the supercapacitor yarns as energy-storing fabrics have also been used to construct a self-charging smart textile through TENG cloth as energy-harvesting fabrics and wearable electronics [225]. TENG cloth is used to harvest the intermittent energy of the human motion, and the converted electrical energy is stored in the supercapacitor yarns as a stable power supply for wearable electronics. Figure 22o shows a wearable micro-cable-structured textile used to simultaneously harvest mechanical and solar energies in order to continuously power an electronic watch [226]. Solar cells are fabricated from lightweight polymer fibers into micro cables. Through a shuttle-flying process, the FiTENGs are woven with micro-cable solar cells in order to construct a smart fabric. The thickness of the smart fabric with single layer is only 320 µm, which ensures that the smart fabric can be easy integrated into various cloths. Under ambient sunlight and in the presence of mechanical excitation, such as wind blowing and human motion, after only 1 min, the hybrid smart textile with a size of 4 cm × 5 cm can charge a 2 mF commercial capacitor up to 2 V. A machine-knitted washable smart textile for accurate epidermal physiological signal monitoring is shown in Figure 22p [227]. It presents a pressure sensitivity of 7.84 mV·Pa^−1^, a response time of 20 ms, a working frequency up to 20 Hz, a stability larger than 100,000 cycles, and machine washability of more than 40 washes. It can be integrated into different parts of clothing to simultaneously monitor respiratory signals and arterial pulse waves. This study provided an efficient, comfortable, and user-friendly approach for detecting human respiration and pulse, representing an advancement in the development of smart textile.

## 5. Summaries and Perspectives

In this review, research progress toward self-powered sensors based on NGs is systematically summarized. Compared with battery technology with limited lifetime, NGs as power sources through harvesting ambient energy are much green and sustainable. Considering the unpredictability of the ambient energy in some special environments, an energy storage unit is necessary to be integrated with the NG. In addition, using NGs to build active sensors can significantly reduce energy consumption. Thus, as effective mechanical-to-electrical signal conversion technologies, PENG and TENG can be used as both active sensing devices and power sources. Self-powered sensors have been successfully utilized in many fields, including physical sensors, wearable devices, biomedical and healthcare applications, human-machine interface, chemical and environmental monitoring, smart traffic, smart cities, robotics, and fiber and fabric sensors. With the tremendous and substantial outcomes already being achieved in those areas, we anticipate significant advances in the near future.

Although significant progress related to self-powered sensors and systems based on NGs has been achieved, there is still a large distance between study and practical commercial applications. Here, potential difficulties and challenges for the extensive commercial applications of TENGs as active sensors and power sources of self-powered sensors and systems are discussed as follows.

### 5.1. Power Sources

*Output performance:* The development TENGs as power sources with high output performance is a constant challenge. According to the more and more mature theoretical studies [21], searching for advanced triboelectric materials and the associated structure design of TENGs are important. We also need to consider how to make sure that a TENG can be perfectly and high effectively integrated into the harvesting environment.

*Durability:* In theory, TENGs can always harvest energy as power sources, such as the TENGs with free standing mode and flexible and stretchable TENGs. However, in many applications, TENGs are made of rigid materials. Abrasion will result during the friction process, which will reduce the performance and lifetime of TENGs. To solve this problem, using the friction between a liquid and a solid body is a good choice. Recently, adding a liquid lubricant between the friction materials has been demonstrated as a good method to reduce abrasion [222].

*Power management:* A power management circuit is a necessary unit to realize a sustained and stable power supply. Due to the output characteristic of TENGs with a large output voltage and a small output current, a matching circuit is a core requirement, which can be studied using some inductance design. A DC-DC circuit, voltage stabilizing circuit, and protection circuit with low consumption are essential. Moreover, a “wake-up” design can be a very useful function of self-powered sensors. The sensors will only send out signals if there is an external trigger. This approach can be used in conjunction with other technologies such as photo-sensing.

*Packaging:* During the operation of TENGs, a large number of electrons are concentrated on the contact layers. It tends to absorb dust in the environment, which will reduce the performance of TENGs. Open-type TENGs may also be invaded by water, which will neutralize electric charges and reduce performance. Hence, a suitable package design without influencing the working of TENGs should be considered.

*Industrial fabrication:* Improving the structure design of TENGs should be done to make sure the component fabrication and assembly can be finished at an industrial scale. This is the necessary work to develop products for wide or commercial use.

### 5.2. Active Sensors

The requirements of durability, packaging, and industrial fabrication are the same as the above. Therefore, we only discuss the future work on sensitivity, stability, and multifunction.

*Sensitivity:* Excellent sensitivity is a constant challenge when developing a sensor. The requirements of output performance listed above also apply to this problem. In addition, some novel structures can also be applied to enhance sensitivity.

*Stability:* Stability is not as important for a sensor with qualitative analysis, but it is one of the most important performance features of a sensor with quantitative analysis. Considering that the output performance of TENGs is strongly related to the environmental parameters such as humidity, some design to keep a stable environment are essential, such as being filled with inert gas or dielectric oil.

*Multifunction:* A sensor with multifunction will reduce the volume and the energy consumption of self-powered sensors. Therefore, this still needs to be considered in the future.
